# Environmental Stochasticity Drives Adaptation to Cooler Thermal Optima in Competition

**DOI:** 10.1007/s11538-026-01614-6

**Published:** 2026-03-18

**Authors:** Abdulrahaman Lawal Suleiman, Pietro Landi, Cang Hui

**Affiliations:** 1 Biomathematics Unit, Department of Mathematical Sciences, Stellenbosch University, Stellenbosch, 7602 South Africa; 2 Department of Mathematics, Federal University Dutse, Dutse, Jigawa State Nigeria; 3 National Institute for Theoretical and Computational Sciences, Stellenbosch University, Stellenbosch, 7602 South Africa; 4 African Institute of Mathematical Sciences, Cape Town, 7945 South Africa

**Keywords:** Competition TPC, Environmental stochasticity, Optimal temperature, Performance breadth, Stochastic Ricker model, Thermal specialisation

## Abstract

Thermal reaction norms, or thermal performance curves (TPCs), describe how ectothermic organisms respond to temperature variation. Here, we investigate how stochastic environmental noise, by modulating competitive interactions among individuals with differing TPC shapes, can drive long-term evolutionary changes in thermal performance. We develop a Ricker competition model within the framework of adaptive dynamics to examine how environmental variability, corresponding to the joint effect of both periodic and stochastic temperature fluctuations, influences the evolution of competition TPCs. Growth, carrying capacity and competitive interactions are all temperature-dependent, with competition coefficients defined by the ratio of individuals’ thermal performance curve at the prevailing temperature. Competition TPCs are modelled using a beta probability density function. Previous results from periodically (i.e., deterministically) fluctuating environments established that the thermal optimum converges to the mean environmental temperature, while performance breadth collapses under constant conditions. In contrast, we show that under stochastic environments, increasing thermal noise broadens competition TPCs and shifts the thermal optimum leftward, away from the environmental mean. This results in right-skewed competition TPCs and suggests an evolutionary bias towards cooler temperature optima for competition under moderate to high environmental noise. Alongside the broadening of competition TPCs to buffer against thermal fluctuations, the shift towards cooler optima reflects a more conservative thermal strategy for competition performance in the face of environmental uncertainty.

**Supplementary Information:**

The online version contains supplementary material available at 10.1007/s11538-026-01614-6.

## Introduction

Temperature is a key environmental factor shaping ectotherm performance, primarily through its effects on physiological rate processes such as fecundity, growth, and metabolism (Angilletta 2009; Mallard et al. 2020; Schulte et al. 2011). Thermal reaction norms, or thermal performance curves (TPCs), capture the responses of these biological rates to ambient temperature change (Schulte et al. 2011). TPCs are typically represented by a unimodal, concave-down function that is often right-skewed, reflecting the nonlinear nature of temperature responses (Angilletta 2009; Schulte et al. 2011; Sinclair et al. 2016). The peak of the curve defines the thermal optimum, while the breadth indicates the organism’s thermal tolerance range, bounded by critical minimum and maximum temperatures (Huey and Kingsolver 1989; Sinclair et al. 2016; Stevenson et al. 1985).

Evolutionary adaptation can modify the thermal optimum, resulting in horizontal shifts in the TPC, and/or alter its breadth under a trade-off between the breadth and height, which influences how effectively organisms perform across their thermal tolerance range (Angilletta 2009; Izem and Kingsolver 2005; Schulte et al. 2011). This performance trade-off can drive the evolution of thermal specialists and generalists (Angilletta 2009). Thermal generalists often evolve in environments characterised by frequent and predictable temperature fluctuations (Buckling et al. 2007; Gilchrist 1995; Kassen 2002). In such contexts, the thermal optimum may either align with or deviate from the mean environmental temperature (Angilletta 2009; Martin and Huey 2008), as shown across a range of empirical and theoretical studies (Amarasekare and Johnson 2017; Gilchrist 1995; Schaum et al. 2022; Suleiman et al. 2025).

As natural environments fluctuate, organisms must evolve adaptive strategies to cope with changing conditions. These strategies include specialisation in constant or near-constant environments, and generalisation in the face of frequent environmental variability. Within this generalist–specialist framework (Gilchrist 1995; Levins 1968), both theoretical and empirical studies on thermal niche evolution have shown that key shape parameters of thermal performance curves (TPCs), such as the thermal optimum (mean) and performance breadth (variance), can underpin the evolution of generalist or specialist strategies under predictably fluctuating conditions (Amarasekare and Johnson 2017; Malusare et al. 2023; Schaum et al. 2022; Suleiman et al. 2025). Along environmental gradients, species’ thermal niche breadths often differ: those inhabiting temperate or polar environments, characterised by colder and more variable climates, tend to be thermal generalists with broader niches than species from warmer, more stable tropical regions (Araújo et al. 2013; Hoffmann et al. 2013; Sexton et al. 2017). However, despite these insights, no theoretical study has yet explored how environmental stochasticity, particularly that arising from random [thus unpredictable] temperature fluctuations, shapes the evolutionary dynamics of TPCs, including shifts in thermal optima and performance breadth.

Building on a previous theoretical work on the adaptation of competition TPCs in constant and predictably fluctuating environments (Suleiman et al. 2025), we investigate how environmental stochasticity and density-dependent competition among individuals with varying competition TPC shapes influence evolutionary dynamics in a population of ectotherms. We adopt the adaptive dynamics (AD) framework, a widely used approach for studying the long-term evolution of life-history traits, in which ecological and evolutionary processes interact to determine outcomes of trait-mediated, density-dependent ecological interactions (Dieckmann and Law 1996; Doebeli 2011; Doebeli and Dieckmann 2000; Geritz et al. 1998; Metz et al. 1992; Waxman and Gavrilets 2005). To examine the evolution of competition TPC shape under unpredictable thermal fluctuations, specifically thermal optima and performance breadth in competition, we develop a stochastic Ricker competition model. Consistent with prior theoretical (Amarasekare and Coutinho 2014; Amarasekare and Johnson 2017; Johnson et al. 2016) and empirical studies (Laws and Belovsky 2010; Van Doorslaer et al. 2009), our model assumes that environmental fluctuations influence population dynamics by modulating temperature-dependent growth, carrying capacity and intraspecific competition. We focus on how random temperature variation drives the evolution of competition TPCs, examining how competition in stochastic environments shapes selective pressures, ultimately leading to shifts in competition TPC shape and a mismatch between the thermal optimum for competition and the mean environmental temperature.

## Model and Methods

### Population Dynamics Under Stochastically Fluctuating Temperatures

We model the stochastic temperature fluctuations experienced by ectotherms in unpredictably varying environments as environmental noise, using the following function:

1 $${\tau }_{t}=\frac{{\tau }_{0}+m\cdot \mathrm{sin}\left(\omega t\right)}{1+\mathrm{exp}\left(-{\sigma }_{\epsilon }{X}_{t}\right)},$$

where ${\tau }_{t}$ denotes the environmental temperature at time $t$ ($=\mathrm{0,1},2,\dots$). The numerator captures the predictable component of temperature fluctuation using a discrete sinusoidal function, with ${\tau }_{0}$ as the vertical shift, $m$ the magnitude and $\omega$ the frequency. When $\omega =n\pi$ for $n=\mathrm{0,1},2,\dots$, or when $m=0$, the sinusoidal term becomes zero, reducing the numerator to ${\tau }_{0}$; otherwise, the numerator contributes to periodic (rational $n$) or quasi-periodic (irrational $n$) fluctuations.

The denominator introduces stochasticity, where ${X}_{t}\sim N(\mathrm{0,1})$ is a standard normally distributed random variable, and ${\sigma }_{\epsilon }$ determines the magnitude of environment noise. This stochastic term is incorporated via a sigmoid transformation, $1/(1+\mathrm{exp}(-{\sigma }_{\epsilon }{X}_{t}))$ (see Fig. S1a–d), which maps the unbounded normal variable to a bounded contribution to temperature (see Appendix S1). When ${\sigma }_{\epsilon }=0$, the temperature simplifies to a deterministic sinusoidal wave, ${\tau }_{t}={(\tau }_{0}+m\cdot \mathrm{sin}\left(\omega t\right))/2$ (Fig. S3a, c). Without loss of generality, we set ${\tau }_{0}$​ and $m$ such that ${(\tau }_{0}-m)>0$ and $({\tau }_{0}+m)<1$, ensuring that ${\tau }_{t}$ remains bounded within (0, 1). For ${\sigma }_{\epsilon }>0$, the temperature dynamics exhibit stochastic fluctuations also strictly within the interval (0, 1) (Fig. S3b, d). Over the long term ($t\to \infty$), the temperature has an expected value of approximately ${\tau }_{0}/2$. The standard deviation of ${\tau }_{t}$ increases with both $m$ and ${\sigma }_{\epsilon }$ (Fig. S3b, d, S4, S5e, f). In this study, we focus on the role of stochastic fluctuations, as the effects of thermal periodicity have been investigated previously (Suleiman et al. 2025).

The discrete-time dynamics of a population with size ${N}_{t}$ are modelled using a stochastic version of the Ricker (1954) model:

2 $$ N_{t + 1} = N_{t} {\mathrm{exp}}\left( {r\left( {\tau_{t} } \right)\left( {1 - \frac{{N_{t} }}{{k\left( {\tau_{t} } \right)}}} \right)} \right), $$

where stochastic temperature fluctuations modulate the intrinsic population growth rate $r\left({\tau }_{t}\right)$ and the carrying capacity $k\left({\tau }_{t}\right)$. This model assumes that environmental stochasticity influences population dynamics by altering demograhic rates (growth and carrying capacity), while the population size is sufficiently large to neglect demographic stochasticity (Ripa and Dieckmann 2013; Ripa and Lundberg 1997).

In line with the metabolic theory of ecology (Brown et al. 2004; Savage et al. 2004), the metabolic rates of ectotherms, and by extension, demographic parameters, exhibit a nonlinear relationship with temperature. Accordingly, the intrinsic growth rate is represented by a saturating exponential function (Fig. S6):

3 $$r({\tau }_{t})={r}_{0}\text{ exp}\left(-\frac{c}{{\tau }_{t}}\right)+{r}_{1},$$

where ${r}_{0}$ is the scaling constant for the growth rate, $c$ controls the steepness of the temperature response, and ${r}_{1}$ denotes the baseline growth rate at near zero temperature. All parameters (${r}_{0}$, $c$ and ${r}_{1}$) are assumed to be real and positive. According to the metabolic theory of ecology and the temperature-size rule (Bernhardt et al. 2018; Savage et al. 2004), the population density of ectotherms at carrying capacity declines with rising temperature. Accordingly, we modelled the carrying capacity $k({\tau }_{t})$ of ectotherms under varying thermal regimes using a bell-shaped function (Fig. S7),

4 $$ k\left( {\tau_{t} } \right) = k_{0} \exp \left( { - \frac{{\left( {\tau_{t} - \tau_{m} } \right)^{2} }}{{\sigma_{k} }}} \right), $$

where ${k}_{0}$ is the scaling constant for carrying capacity, ${\tau }_{m}$ is the environmental temperature at which carrying capacity peaks, and ${\sigma }_{k}$ determines the breadth of the temperature response. Maximum carrying capacity (${k}_{0}$) is attained at an intermediate environmental temperature (${\tau }_{m}=0.5$), bounded by the critical thermal minimum ${\mathrm{C}\tau }_{\mathrm{min}}$ and the critical thermal maximum ${\mathrm{C}\tau }_{\mathrm{max}}$, setting to 0 and 1 respectively. The bell-shaped carrying capacity thus also assumes that the species can maintain only a small population size under extremely low temperatures. These temperature-dependent, nonlinear functions of performance and fitness components represent the TPCs of demographic rates (in particular, growth rate and carrying capacity).

### Resident-Mutant Population Dynamics

To model the adaptation of competition TPCs in stochastically fluctuating environments, we assume that rare mutants (${M}_{t}$) occasionally arise within a resident population (${N}_{t}$) at time $t$, with mutant competition TPC shapes differing slightly from those of the residents. The functional forms of the intrinsic growth rate and carrying capacity remain identical between residents and mutants. The coupled dynamics of the resident and mutant populations are modelled using the intraspecific Ricker competition model:

5 $$ \begin{aligned} N_{t + 1} & = N_{t} {\mathrm{exp}}\left( {r\left( {\tau_{t} } \right)\left( {1 - \frac{{N_{t} + \alpha_{NM} \left( {\tau_{t} } \right)M_{t} }}{{k\left( {\tau_{t} } \right)}}} \right)} \right) \\ M_{t + 1} & = M_{t} {\mathrm{exp}}\left( {r\left( {\tau_{t} } \right)\left( {1 - \frac{{\alpha_{MN} \left( {\tau_{t} } \right)N_{t} + M_{t} }}{{k\left( {\tau_{t} } \right)}}} \right)} \right) \\ \end{aligned} , $$

where ${\alpha }_{MN}({\tau }_{t})$ represents the temperature-dependent competitive effect of residents on mutants, and ${\alpha }_{NM}({\tau }_{t})$ denotes the reciprocal effect of mutants on residents. Both competition coefficients depend on the prevailing stochastic temperature ${\tau }_{t}$, which captures the environmental noise.

We assume incremental evolution, where mutant and resident individuals respond demographically to the thermal environment in an identical manner. That is, the population-level demographic traits (including the intrinsic growth rate and carrying capacity) of the rare mutants exhibit the same temperature dependence as those of the resident population. The mutation introduces only a minute difference in individual-level competition performance during resource contests between mutants and residents, allowing for relative differentiation in their competitive abilities. Accordingly, the model assumes that the thermal performance curve (TPC) for competitive performance does not affect the intrinsic growth rate and carrying capacity (although both remain temperature dependent) but affects only the intensity of intraspecific competition between residents and mutants of the same species. The performance traits therefore represent those relevant to individual-level resource competition, typically measurable in the field and/or physiological laboratory (e.g., speed, motility, aggression and feeding rate), as compared to the population-level demographic traits (intrinsic growth rate and carrying capacity) that would require population-level survey of demographics.

Specifically, the competition coefficients are calculated as the ratio of competition TPCs evaluated at the current environmental temperature (Schoener 1974; Suleiman et al. 2025; Appendix S2),

6 $$ \begin{aligned} \alpha_{MN} \left( {\tau_{t} } \right) & = \frac{{\beta_{N} \left( {\tau_{t} ;\mu_{N} , s_{N} } \right)}}{{\beta_{M} \left( {\tau_{t} ;\mu_{M} , s_{M} } \right)}}, \\ \alpha_{NM} \left( {\tau_{t} } \right) & = \frac{{\beta_{M} \left( {\tau_{t} ;\mu_{M} , s_{M} } \right)}}{{\beta_{N} \left( {\tau_{t} ;\mu_{N} , s_{N} } \right)}}, \\ \end{aligned} $$

where ${\beta }_{x}\left({\tau }_{t};{\mu }_{x},{s}_{x}\right)$ denotes the beta probability density function representing the competition TPC of type $x$ (resident $N$ or mutant $M$), with mean ${\mu }_{x}\in (\mathrm{0,1})$ and scaling parameter ${s}_{x}\in (0,\infty )$, which determines the breadth of the curve. When ${\mu }_{N}={\mu }_{M}$ and ${s}_{N}={s}_{M}$, the two competition TPCs are identical (${\beta }_{N}\left({\tau }_{t}\right)={\beta }_{M}\left({\tau }_{t}\right)$), and thus ${\alpha }_{NN}\left({\tau }_{t}\right)={\alpha }_{MM}\left({\tau }_{t}\right)=1$, reflecting the competitive effects experienced by identical individuals of the same species. When the competition TPC shapes differ between the resident and mutant types (${\beta }_{N}\left({\tau }_{t}\right)\ne {\beta }_{M}\left({\tau }_{t}\right)$), the competition coefficients become asymmetric (${\alpha }_{MN}\left({\tau }_{t}\right)=1/{\alpha }_{NM}\left({\tau }_{t}\right)\ne 1$). Consequently, at a given temperature ${\tau }_{t}$, one type performs better than the other, resulting in unequal competition, i.e., competitive asymmetry.

**Fig. 1:**
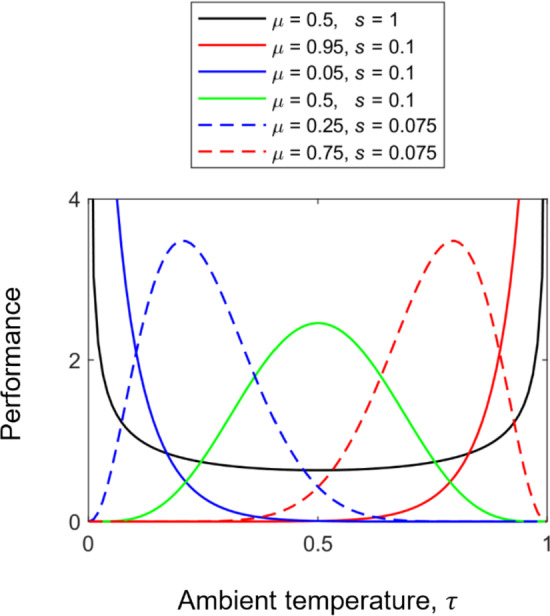
Plots of the beta distribution function, $\beta (\tau ;\mu ,s)$. The curves describe the competitive performance at the ambient temperature ($\tau$), for different values of the mean ($\mu$) and scaling parameter ($s$). The curve can be bimodal U-shaped (black curve), monotonic right- or left-skewed (blue and red dashed curves, respectively), right- or left-skewed J-shaped (blue and red solid curves, respectively), and also symmetric (green curve). The unimodal curves can also be used to describe the specialist-generalist trade-off associated with the variation in performance curve shapes. A cold (blue curves) or heat specialist (red curves) will have a higher maximal performance than a thermal generalist (green curve). This trade-off is built in the beta distribution function since the area under the performance curve is conserved (Angilletta 2009) (Color figure online)

To explore how the competition TPCs evolve in response to stochastic temperature fluctuations, we analyse how changes in two key parameters of the thermal regime—environmental noise magnitude (${\sigma }_{\epsilon }$) and fluctuation frequency ($\omega$) under different fixed sine magnitude ($m$)—affect the evolutionary outcomes for the competition TPC’s optimal temperature (${\tau }_{\mathrm{opt}}$) and breath ($\sigma$). While ${\tau }_{\mathrm{opt}}$ and $\sigma$ are derived from the shape parameters $\mu$ and $s$, they are not identical. Specifically, ${\tau }_{\mathrm{opt}}=\left(\mu -s\right)/\left(1-2s\right)$ and $\sigma =\sqrt{s\mu \left(1-\mu \right)/\left(s+1\right)}$. Generally, the definition of the optimum temperature depends on the temperature-dependent performance trait considered. In physiological and ecological studies of insect TPCs (e.g., Angilletta 2009; Amarasekare and Savage 2012; Deutsch et al. 2008; Schulte et al. 2011), performance traits are diverse and primarily include locomotor performance (e.g., walking or flight speed, jump distance, righting response time, motility, foraging efficiency, predator avoidance, and competitive ability), as well as developmental and life-history traits (e.g., development rate, time to maturity, fecundity, and the intrinsic rate of increase $r$, which is typically derived rather than directly measured). Other commonly examined traits include survival and mortality metrics, and feeding or metabolic performance. Here, the optimum temperature refers explicitly to the temperature at which competitive performance is maximised, that is, the peak of the thermal performance curve (TPC) for competition (Fig. 1), and not to the temperature that maximises the intrinsic or per-capita population growth rate, or the carrying capacity (see Discussion). In the next section, we model the evolution of competition TPC shapes (i.e., $\mu$ and $s$) and use these formulas to compute ${\tau }_{\mathrm{opt}}$ and $\sigma$.

### Evolutionary Dynamics

To investigate the adaptation of competition TPCs under stochastic thermal conditions, we employ the adaptive dynamics (AD) framework (Dieckmann and Law 1996; Geritz et al. 1998; Metz et al. 1992). Within this framework, the evolutionary rate of change of any trait is proportional to the selection gradient acting on that trait. The selection gradient is defined as the partial derivative (slope) of mutant (or invasion) fitness with respect to the focal trait, evaluated at the resident trait value and under the assumption that the mutant population size is infinitesimally small. Mutant fitness can be interpreted as the per-capita growth rate of the mutant in a resident population at ecological equilibrium (where, by definition, the per-capita growth rate of the resident is zero). Expressing selection in terms of the difference in growth rates between mutant and resident types is also valid. Because the mutant population is assumed to be vanishingly rare, mutant–mutant interactions are excluded from the calculation of mutant fitness. The following and the Supplementary Information provide the full mathematical formulation underlying these definitions.

The competition TPC shape is characterised by two parameters: the mean ($\mu$) and scaling parameter ($s$). In a fluctuating environment, the invasion fitness of a rare mutant is defined as the long-term average of its per-capita growth rate when introduced at low abundance (${M}_{t}\to 0$) into a resident population at its stationary distribution (Metz et al. 1992). This is computed as follows:

7 $$\overline{\lambda }\left({\mu }_{N},{s}_{N}, {\mu }_{M},{s}_{M}\right)=\underset{L\to \infty }{\mathrm{lim}}\frac{1}{L}\sum_{t=0}^{L}\left(r({\tau }_{t})\left(1-\frac{{\alpha }_{MN}({\tau }_{t}){N}_{t}}{k({\tau }_{t})}\right)\right),$$

where $L$ is the time horizon over which the average is taken. The invasion fitness $\overline{\lambda }$ depends on the competition TPC shape of both the resident and mutant populations and the population size of the resident.

The direction of evolution is governed by the selection gradients, defined as the partial derivatives of the invasion fitness with respect to the mutant trait values, evaluated at the resident trait values (see Appendix S3):

8 $$ \begin{aligned} \overline{g}_{\mu } & = \left. {\frac{{\partial \overline{\lambda }\left( {\mu_{N} ,s_{N} , \mu_{M} ,s_{M} } \right)}}{{\partial \mu_{M} }}} \right|_{{\left( {\mu_{M} , s_{M} } \right) = \left( {\mu_{N} , s_{N} } \right)}} , \\ \overline{g}_{s} & = \left. {\frac{{\partial \overline{\lambda }\left( {\mu_{N} ,s_{N} , \mu_{M} ,s_{M} } \right)}}{{\partial s_{M} }}} \right|_{{\left( {\mu_{M} , s_{M} } \right) = \left( {\mu_{N} , s_{N} } \right)}} . \\ \end{aligned} $$

Repeated cycles of mutation, invasion, and substitution drive the resident traits along the direction of these selection gradients. A positive ${\overline{g} }_{\mu }$ implies selection for higher TPC mean (i.e., a shift toward stronger competition in warmer temperatures), whereas a negative gradient favours lower means. Similarly, ${\overline{g} }_{s}>0$ promotes the evolution of broader competition TPCs, while ${\overline{g} }_{s}<0$ favours narrower curves.

Evolution proceeds until it reaches an evolutionary singularity $\left({\mu }^{*},{s}^{*}\right)$, a point where both selection gradients vanish. The stability of this singularity is determined by the Hessian matrix ${H}^{*}$, which contains the second derivatives of the invasion fitness with respect to mutant traits, evaluated at the singularity. The definiteness of ${H}^{*}$ determines whether the singularity is an evolutionarily stable strategy (ESS) or a potential branching point under disruptive selection (Della Rossa et al. 2015; Dercole et al. 2016; Geritz et al. 2016) (Appendix S4). We computed evolutionary nullclines, where ${\overline{g} }_{\mu }=0$ and ${\overline{g} }_{s}=0$, and located their intersection, which defines the evolutionary singularity (${\mu }^{*},{s}^{*}$).

**Fig. 2:**
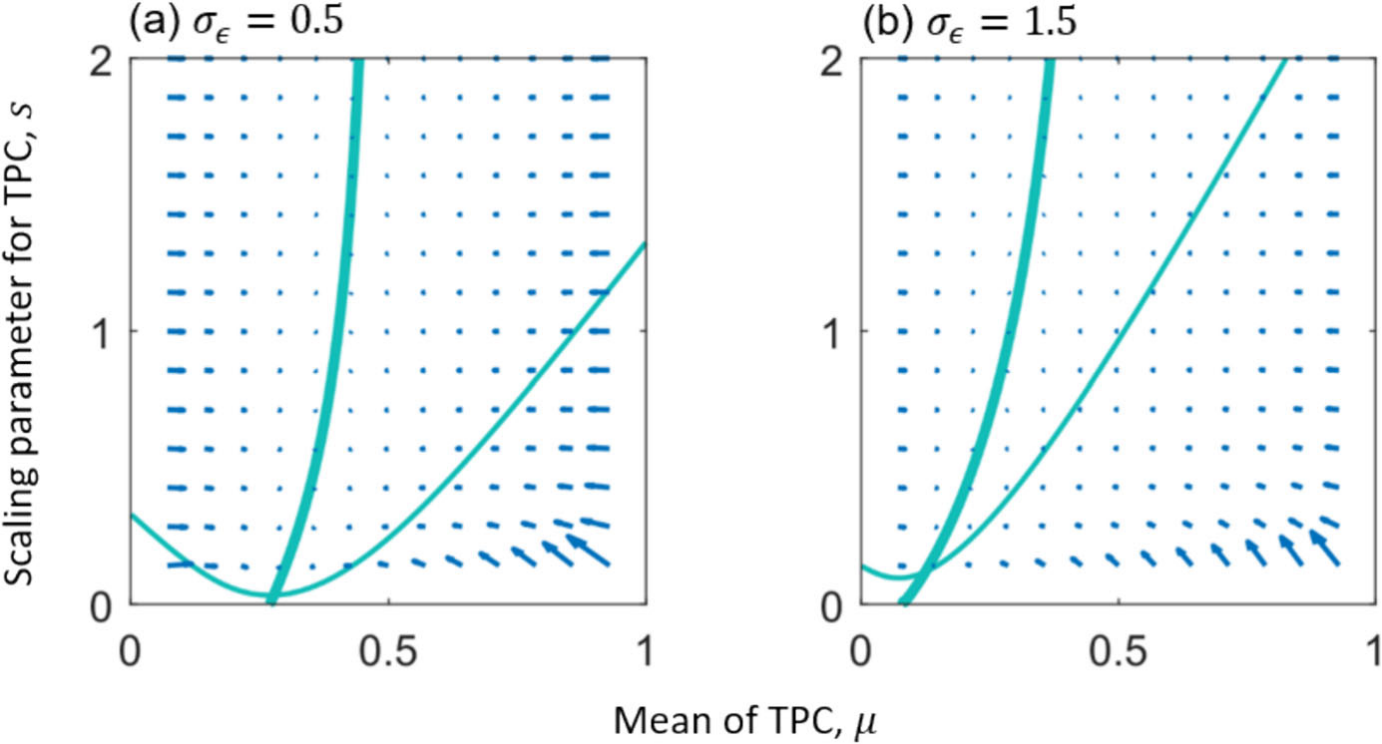
Evolutionary vector fields of the shape parameters of competition TPCs in Eq. (9). Arrows show the magnitude and direction of evolutionary change. Bold curves represent the nullcline for the selection gradient of the TPC mean (${\overline{g} }_{\mu }=0$), and thin curves represent the nullcline for the TPC scaling parameter (${\overline{g} }_{s}=0$). Singular points are numerically computed as the intersection of the nullclines with (a) $({\mu }^{*},{s}^{*})=(\mathrm{0.28,0.04})$ for low-magnitude noise (${\sigma }_{\epsilon }=0.5$) and (b) $({\mu }^{*},{s}^{*})=(\mathrm{0.13,0.13})$ for high-magnitude noise (${\sigma }_{\epsilon }=1.5$). All singularities are continuously stable (convergence stable, meaning the evolutionary dynamics converge towards them, and evolutionarily stable, indicating the final stop of the evolutionary process). In these figures, the mutational covariance matrix $\mathrm{E}$ is set to the identity matrix. Parameters: ${r}_{0}=c={r}_{1}=0.1$, ${k}_{0}=2$, ${\tau }_{0}=0.5$, $m=0.25$, $\omega =13\pi /20$, and ${\sigma }_{k}=0.05$. The list of model parameters is provided in Table 1

We assessed the convergence stability of this singular point by examining the 2-dimensional evolutionary vector field in the ($\mu$, $s$) plane (Fig. 2), and further verified it by computing the eigenvalues of the Jacobian matrix derived from the canonical equations $\dot{\mu }=\frac{1}{2}{\widehat{N}}_{e}\mathrm{E} {\overline{g} }_{\mu }$ and $\dot{s}=\frac{1}{2}{\widehat{N}}_{e}\mathrm{E} {\overline{g} }_{s}$, where ${\widehat{N}}_{e}$ depicts the long-term average effective population size, the constant 1/2 indicates the fraction of mutations lost due to directional selection, $\mathrm{E}$ is the 2 × 2 mutational covariance matrix (Geritz et al. 2016), which represents the mutational dependence of the two TPC shape parameters. To compute the singular points of the dynamical system, we set $\mathrm{E}$ to the identity matrix (independent mutations in $\mu$ and $s$). The results are shown in Fig. 2. We further show that the results are robust under random mutational covariance since the nullclines of the dynamical system computed using the identity and random mutational covariance matrices intersect at the same evolutionary singularity (Fig. S11) and have the same convergence stability (Fig. S12). Additionally, we evaluated the evolutionary stability by calculating the eigenvalues of the Hessian matrix at the singularity (see Appendix S4). Parameter values used for these simulations are provided in Table 1.

**Table 1:** Summary of the model parameters and their description

Parameter	Description	Reference value/range
${r}_{0}$	Scaling constant for the growth rate	${r}_{0}=0.1, 5$
$c$	Steepness of the growth response	$c=0.1$
${r}_{1}$	Baseline growth rate at near zero temperature	${r}_{1}=0.1$
${k}_{0}$	Scaling constant for the carrying capacity	${k}_{0}=1, 2$
${\sigma }_{k}$	Width of carrying capacity	${\sigma }_{k}=0.05$
${\tau }_{m}$	Optimal temperature of carrying capacity	${\tau }_{m}=0.5$
${\tau }_{0}$	Average ambient temperature	${\tau }_{0}=0.5$
$m$	Sine magnitude of temperature fluctuations	$m=0.25, 0.4$
$\omega$	Frequency of temperature fluctuations	$\omega \in (\mathrm{0,2}\pi )$
${\sigma }_{\epsilon }$	Environmental noise magnitude	${\sigma }_{\epsilon }\in (\mathrm{0,2})$
$\mu$	Mean of performance curve	$\mu \in (\mathrm{0,1})$
$s$	Scaling parameter for the performance curve	$s\in (\mathrm{0,2})$
${\tau }_{\mathrm{opt}}$	Optimal temperature of performance curve	${\tau }_{\mathrm{opt}}=\left(\mu -s\right)/\left(1-2s\right)$
$\sigma$	Breath of performance curve	$\sigma =\sqrt{s\mu \left(1-\mu \right)/\left(s+1\right)}$

## Results

### Population Dynamics Under Stochastic Temperature Fluctuations

**Fig. 3:**
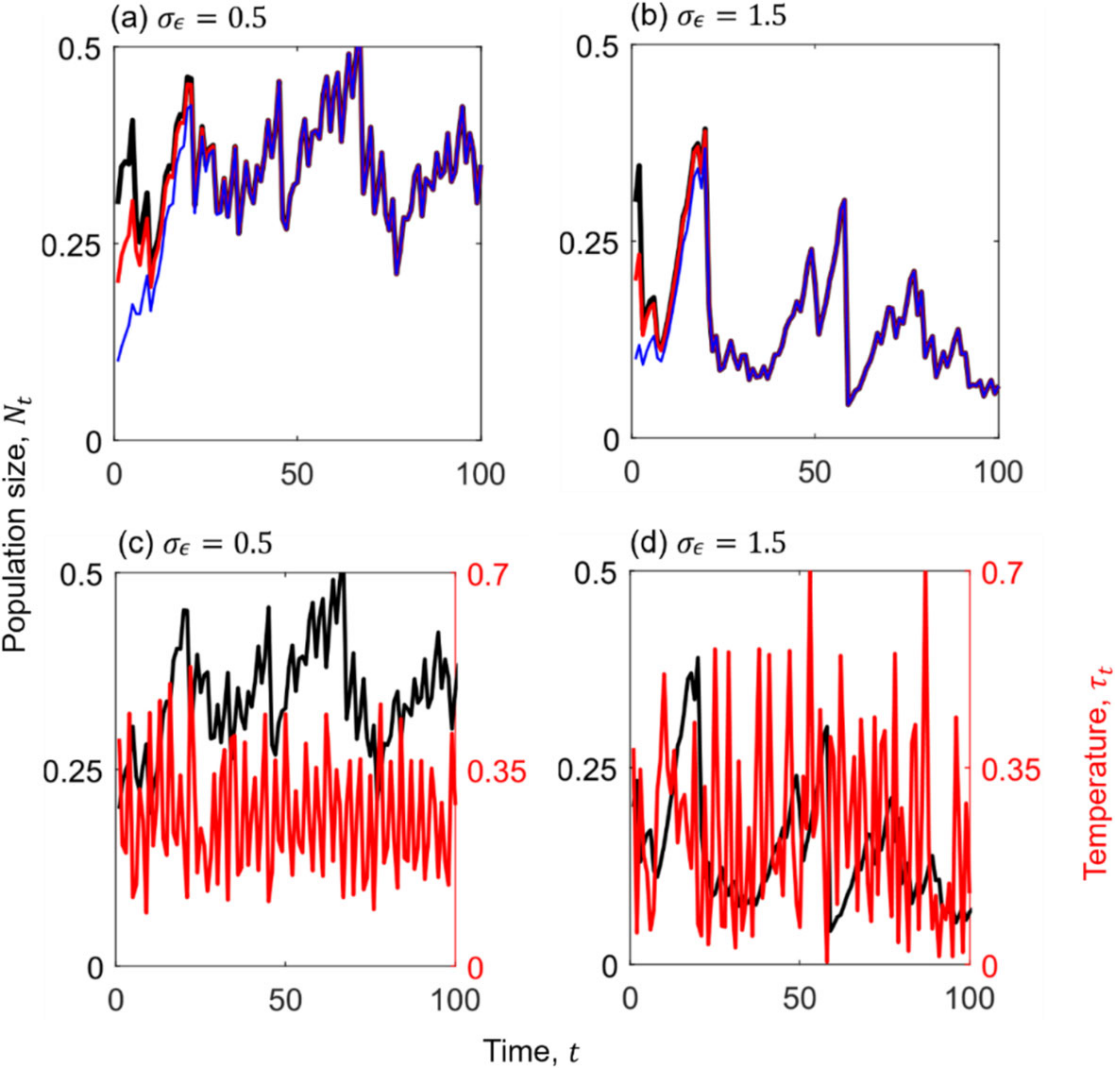
Effects of stochastic temperature fluctuations (Eq. 1) on resident population dynamics (Eq. 2). **a** and **b** Population dynamics from three different initial conditions (${N}_{0}=0.3$, 0.2, $0.1$; shown in black, red and blue, respectively) converge to the same long-term oscillating behaviour under low (**a**) and high (**b**) environmental noise. Initial conditions lose influence within the first 100 timesteps. **c** and **d** Stochastic temperature dynamics (red line; right y-axis) under low (**c**, ${\sigma }_{\epsilon }=0.5$) and high (**d**, ${\sigma }_{\epsilon }=1.5$) noise magnitudes. Corresponding population dynamics (black lines, left y-axis) show higher values under low-magnitude noise (**c**) than under high-magnitude noise (**d**). Model parameters: ${r}_{0}=c={r}_{1}=0.1$, ${k}_{0}=2$, ${\tau }_{0}=0.5$, $m=0.25$, $\omega =13\pi /20$, and ${\sigma }_{k}=0.05$. The list of model parameters is provided in Table 1 (Color figure online)

Without considering mutation or adaptive evolution, fluctuations in environmental temperature under different parameter settings in Eq. (2) influence the intrinsic growth rate and carrying capacity, which in turn drive temporal changes in population size ${N}_{t}$. Without environmental noise (i.e., ${\sigma }_{\epsilon }=0$), the population dynamics can still exhibit different ecological behaviours (regular steady-state, periodic, and chaotic; Figs. S1a–b, c–d, and e–f, respectively). Under a specific sequence of environmental noise (${\sigma }_{\epsilon }>0$), population dynamics under the same parameter values consistently converge to the same aperiodic oscillation attractor after a brief transient period (Fig. 3a, b; Fig. S1g), regardless of initial population sizes. Notably, the resulting aperiodic oscillation attractors do not directly track the pattern of environmental temperature fluctuations (Fig. 3c, d; Fig. S1h). As expected, higher environmental noise magnitudes lead to greater variability in temperature. For instance, the temperature profile under high (${\sigma }_{\epsilon }=1.5$; Fig. 3d) and intermediate noise magnitude (${\sigma }_{\epsilon }=1$; Fig. S1h) displays pronounced fluctuations compared to the more stable regime under low noise magnitude (${\sigma }_{\epsilon }=0.5$, Fig. 3c). These temperature-driven fluctuations propagate through the ecological dynamics, generating aperiodic fluctuations in population size. Populations subjected to lower environmental noise magnitude (${\sigma }_{\epsilon }=0.5$) tend to reach higher densities (Fig. 3a, c) than those under intermediate (${\sigma }_{\epsilon }=1$; Fig. S1g) or high noise magnitude (${\sigma }_{\epsilon }=1.5$; Fig. 3b, d), reflecting disruptive effects of high thermal variability on demographic performance.

### Evolutionary Singularity Under Stochastic Temperature Fluctuations

When environmental noise is absent (${\sigma }_{\epsilon }=0$), temperature fluctuations follow a deterministic discrete (quasi-)periodic pattern with an average of ${\tau }_{0}/2$. When the frequency of the sinusoidal fluctuation $\omega$ is set to $2\pi$, temperatures become constant across integer time steps. Under these two thermal regimes, the evolutionary singularity of the competition TPC, defined by $\left({\mu }^{*},{s}^{*}\right)$, constitutes a continuously stable strategy (CSS; Eshel 1983), which is both convergence stable and evolutionarily stable (Appendix S4). At this singularity, when the temperature is constant (period 1), the optimal temperature of the competition TPC (${\tau }_{\mathrm{opt}}$) aligns with the average environmental temperature (${\tau }_{0}/2$), and the performance breadth ($\sigma$) shrinks to zero (Fig. S2). When the temperature is not constant, the evolutionary singularity shifts horizontally toward lower temperatures (${\tau }_{\mathrm{opt}}<{\tau }_{0}/2$) and the breadth of the competition TPC increases.

Under stochastic thermal regimes (${\sigma }_{\epsilon }>0$), ambient temperature fluctuates randomly, leading to stochastic variation in the intrinsic population growth rate (Fig. S8) and carrying capacity (Fig. S9). This variation causes the evolutionary singularity to shift horizontally toward lower temperatures (${\tau }_{\mathrm{opt}}<{\tau }_{0}/2$), and the breadth of the competition TPC increases further with noise magnitude ($\sigma$ increases with ${\sigma }_{\epsilon }$ under fixed $m$; Fig. 2). These shifts reflect adaptive responses to increased thermal uncertainty and variability. All identified singularities remain continuously stable, as confirmed by both the convergence patterns in the selection-gradient vector fields (Fig. 2) and numerical assessments of evolutionary stability (Appendix S4).

To assess the robustness of our results to changes in the parameters affecting the intrinsic growth rate (${r}_{0}$, $c$, and ${r}_{1}$) and carrying capacity (${k}_{0}$ and ${\sigma }_{k}$), as listed in Table 1, we conducted additional sensitivity tests on the parameter pairs (${r}_{0}$ vs ${r}_{1}$, ${r}_{0}$ vs $c$ and ${k}_{0}$ vs ${\sigma }_{k}$). The results confirm that the ESS remains robust to these parameter changes (see Fig. S10).

### Evolutionarily Singular Thermal Performance

**Fig. 4:**
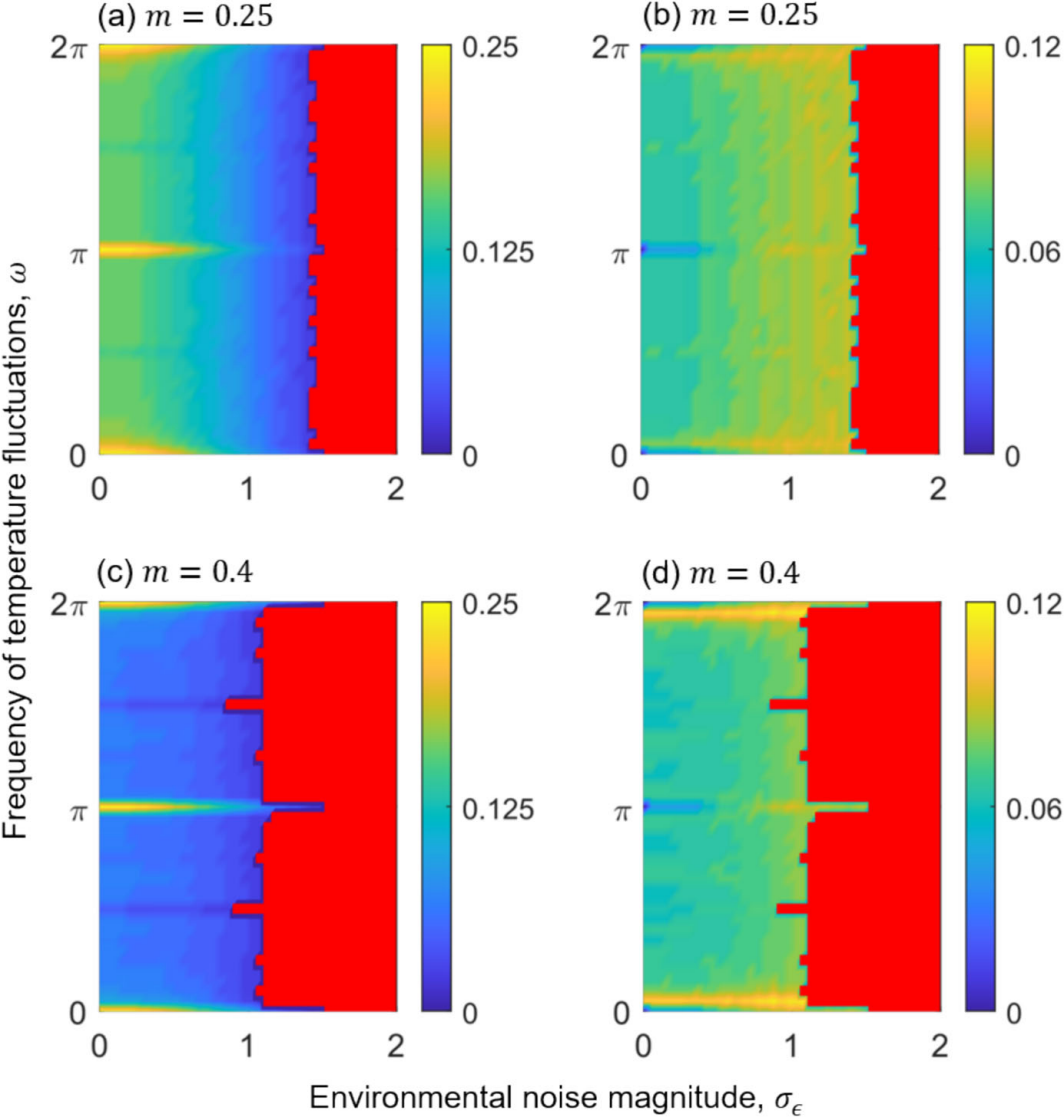
Heat maps of the evolutionarily stable TPC optimal temperature and standard deviation of competitive performance as functions of the environmental noise magnitude (${\sigma }_{\epsilon }$) and the frequency of sinusoidal temperature fluctuations ($\omega$). The TPC optimal temperature (${\tau }_{\mathrm{opt}}$; panels **a**, **c**) and standard deviation ($\sigma$; panels **b**, **d**), for intermediate sine magnitude ($m=0.25$; panels **a**, **b**) and large sine magnitude ($m=0.4$ panels **c**, **d**). The coloured regions indicate shapes of unimodal TPCs, while red regions represent right-skewed J-shaped TPCs. Colour intensity reflects the ESS values of ${\tau }_{\mathrm{opt}}$ (**a**, **c**) and $\sigma$ (**b**, **d**) under selection of corresponding environmental thermal regimes. Parameters: ${r}_{0}=c={r}_{1}=0.1$, ${k}_{0}=2$, ${\tau }_{0}=0.5$, and ${\sigma }_{k}=0.05$. The list of model parameters is provided in Table 1

Our analyses reveal the emergence of two major competition TPC types under stochastic fluctuating environments: unimodal curves (coloured regions in Fig. 4) and right-skewed J-shaped curves (red regions in Fig. 4). When the deterministic component of the temperature fluctuations is constant and the stochastic component is weak (i.e., when $\omega =n\pi ; n=\mathrm{0,1},2,\dots$, and ${\sigma }_{\epsilon }<1$), the optimal temperature (${\tau }_{\mathrm{opt}}$) of the competition TPC aligns with the mean environmental temperature (${\tau }_{0}/2=0.25$; Fig. 4). However, even under low noise, when the frequency of temperature fluctuation becomes non-integer (i.e., a quasi-/periodic), the optimal temperature for competition (${\tau }_{\mathrm{opt}}$) shifts leftward from the mean, indicating a bias toward lower temperatures for peak competitive performance.

Under a moderate sinusoidal magnitude ($m=0.25$), the optimal temperature (${\tau }_{\mathrm{opt}}$) and breadth ($\sigma$) of unimodal TPCs for competition exhibit patterns corresponding to change in temperature periodicity captured by sinusoidal frequency $\omega$ (Fig. 4a, b). As noise magnitude increases, ${\tau }_{\mathrm{opt}}$ declines and $\sigma$ increases. At low noise (${\sigma }_{\epsilon }<1$), ${\tau }_{\mathrm{opt}}$ matches the mean (${\tau_{0}}/{2}=0.25$) at $\omega =0$, $\pi$ and $2\pi$ (where the sine function yields a constant value), but decreases as $\omega$ deviates from these points (Fig. 4a). Accordingly, the breadth $\sigma$ shrinks at these frequencies, corresponding to minimal environmental oscillations due to a zero-valued sine component, and expands when $\omega$ deviates from these frequencies (Fig. 4b). Under higher noise (${\sigma }_{\epsilon }\ge 1$), ${\tau }_{\mathrm{opt}}$ continues to decrease, and $\sigma$ increases across a broad range of $\omega$ values (Fig. 4a, b).

With a higher sinusoidal magnitude ($m=0.4$), similar patterns are observed (Fig. 4c, d). At low noise levels (${\sigma }_{\epsilon }\le 1$), ${\tau }_{\mathrm{opt}}$ again aligns with the environmental mean at $\omega =0$, $\pi$ and $2\pi$, but shifts to even lower temperature when deviating from these frequencies (Fig. 4c). The breadth $\sigma$ shrinks to nearly zero at these frequencies but increases sharply when deviating from these frequencies and settles to intermediate values (Fig. 4d).

Right-skewed J-shaped TPCs for competition arise within a narrow parameter range under moderate sinusoidal magnitude ($m=0.25$) and high environment noise (${\sigma }_{\epsilon }>1.5$; red regions in Fig. 4a, b). Under high sinusoidal magnitude ($m=0.4$), these skewed curves appear more broadly across moderate to large noise levels (${\sigma }_{\epsilon }\ge 1$; Fig. 4c, d). These J-shaped TPCs, characterised by ${\tau }_{\mathrm{opt}}=0$, reflect evolutionary specialisation toward cold extremes, where thermal performance in competition peaks at the lowest environmental temperatures experienced by the population.

**Fig. 5:**
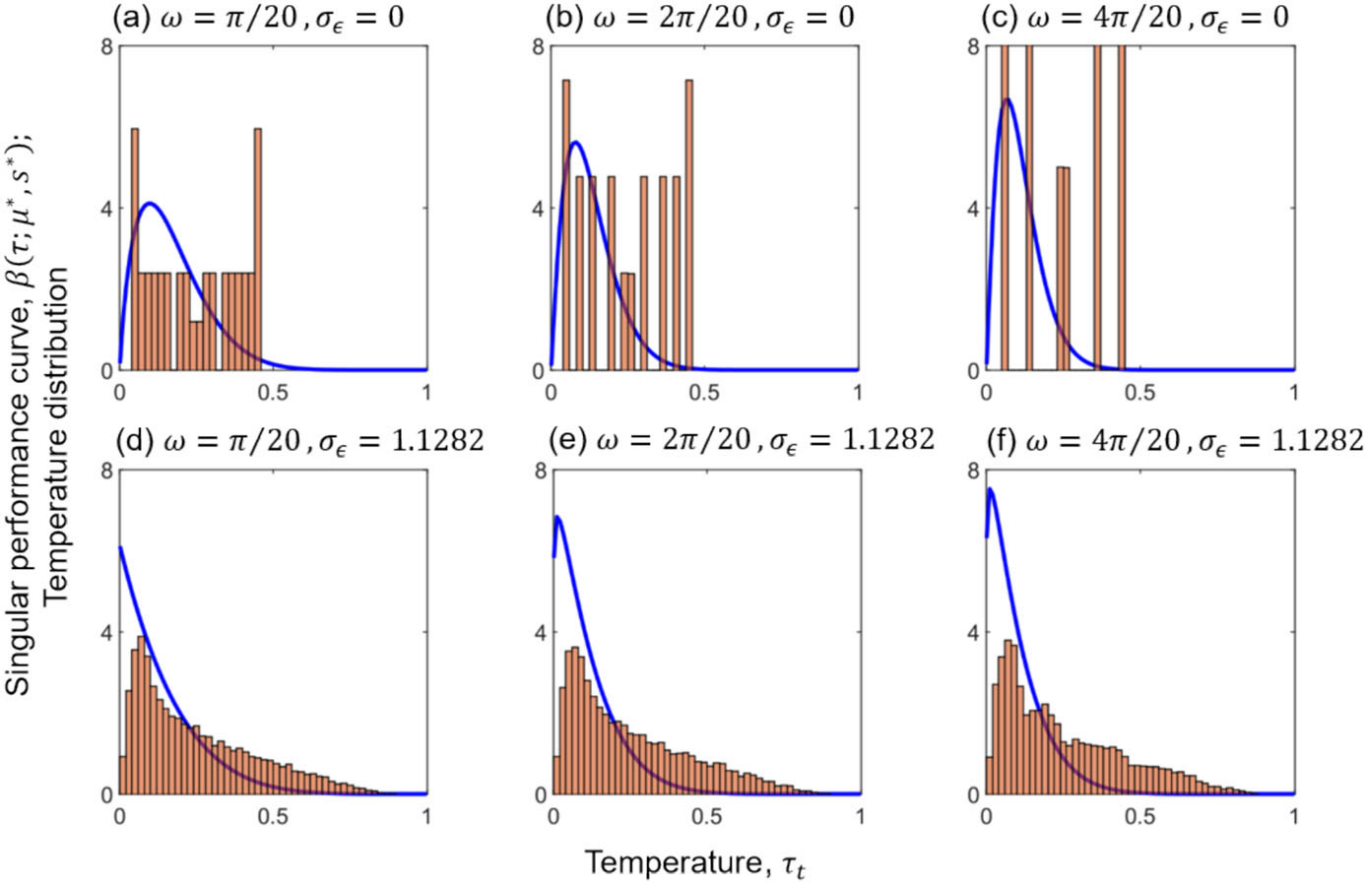
Histogram of the temperature distribution and singular thermal curve for competitive performance (TPC). The temperatures are displayed as normalised probability distributions without environmental noise (${\sigma }_{\epsilon }=0$; plots **a**–**c**) and with environmental noise (${\sigma }_{\epsilon }=1.1282$; plots **d**–**f**). The blue curves depict singular unimodal performance curves $\beta (\tau ;{\mu }^{*},{s}^{*})$ computed as follows: **a** $({\mu }^{*},{s}^{*})=(\mathrm{0.17,0.09})$ for $\omega =\pi /20$ with optimal temperature ${\tau }_{\mathrm{opt}}=0.0976$ and standard deviation $\sigma =0.0117$; **b** $({\mu }^{*},{s}^{*})=(\mathrm{0.13,0.06})$ for $\omega =2\pi /20$ with ${\tau }_{\mathrm{opt}}=0.0795$ and $\sigma =0.0064$; **c** $({\mu }^{*},{s}^{*})=(\mathrm{0.11,0.05})$ for $\omega =4\pi /20$ with ${\tau }_{\mathrm{opt}}=0.0667$ and $\sigma =0.0047$; **d** $({\mu }^{*},{s}^{*})=(\mathrm{0.14,0.14})$ for $\omega =\pi /20$ with optimal temperature ${\tau }_{\mathrm{opt}}=0$ and standard deviation $\sigma =0.0148$; **e** $({\mu }^{*},{s}^{*})=(\mathrm{0.11,0.1})$ for $\omega =2\pi /20$ with ${\tau }_{\mathrm{opt}}=0.0125$ and $\sigma =0.0089$; **f** $({\mu }^{*},{s}^{*})=(\mathrm{0.1,0.09})$ for $\omega =4\pi /20$ with ${\tau }_{\mathrm{opt}}=0.0122$ and $\sigma =0.0074$. Parameters: ${\tau }_{0}=0.5$, $m=0.4$. The list of all model parameters is provided in Table 1 (Color figure online)

The leftward shift in ${\tau }_{\mathrm{opt}}$ relative to the mean (${\tau }_{0}/2=0.25$) is illustrated by normalised histograms of environmental temperature and the corresponding singular reaction norms (Fig. 5). Focusing on stochastic temperature distributions with large sine magnitudes ($m=0.4$), we examined the shapes of evolutionarily singular reaction norms across different frequencies and noise magnitudes. Our findings show that more right-skewed singular TPCs for competitive performance evolve under broader (i.e., more variable) stochastic temperature distributions, whereas less right-skewed unimodal TPCs evolve under narrower, deterministic regimes (compare top and bottom panels of Fig. 5). Right skewness increases with $\omega$ in deterministic environments (top row), while it decreases with $\omega$ in strongly noisy environments (bottom row). The largest right skewness happens in panel d where the competition TPC becomes J-shaped.

## Discussion

### Thermal Competition in Fluctuating Environments

Under quasi-periodic and stochastic environmental temperature fluctuations, our model predicts the evolution of both thermal specialists and generalists in competitive performance, as well as the emergence of unimodal and right-skewed J-shaped competition TPCs. In environments with constant temperatures, or in stochastic regimes with zero sine magnitude and low noise magnitude (Fig. 4), the TPC optimal temperature for competitive performance aligns with the environmental mean, while the competition TPC breadth collapses to close to zero. These steep competition TPCs reflect full thermal specialisation in competitive contests, consistent with prior findings that narrower TPCs (with higher peak performance in competition) evolve under constant or low-variability thermal regimes, whereas greater environmental variability selects for broader performance curves (Amarasekare and Johnson 2017; Suleiman et al. 2025). Building on these insights, our results show that thermal specialists with narrow competition TPCs also evolve under intermediate ($m=0.25$) or high ($m=0.4$) sine magnitude of temperature fluctuations with low environmental noise, even when the optimal temperature for competition deviates to lower than the mean temperature (${\tau }_{0}/2$). Such moderately narrow TPCs, indicative of specialisation in competitive performance, evolve under low-magnitude stochastic fluctuations (${\sigma }_{\epsilon }<0.5$), whereas broader TPCs, characteristic of thermal generalists in competition, emerge under intermediate to high noise levels (${\sigma }_{\epsilon }\ge 0.5$), with optimal temperatures for peak competitive performance consistently shifted below the mean temperature (Fig. 4). Notably, in the absence of stochastic fluctuations and when temperature fluctuation frequencies align with $\omega =n\pi$ (where $n$ is an integer), TPC optimal temperatures for competition remain aligned with the mean (Fig. 4). In contrast, fluctuations with non-integer harmonic frequencies drive optimal temperatures below the mean (Fig. 4) even without stochastic fluctuations. These findings align with prior research showing that fluctuating thermal regimes tend to favour thermal generalists, while stable environments select for specialists (Buckling et al. 2007; Duncan et al. 2011; Kassen 2002; Kassen and Bell 1998; Schaum et al. 2022; Suleiman et al. 2025).

When TPC optimal temperatures for competition evolve below the environmental mean, the TPC breadth increases, showing a clear monotonic relationship across stochastic thermal regimes (Fig. 4b, d). Specifically, as stochastic fluctuations drive the optimal temperature for peak competition leftward of the mean, TPC breadth expands; conversely, when optimal temperatures approach the mean, competition TPCs become narrower (Fig. 4). These patterns are consistent with theoretical and empirical studies showing that under strong but predictable fluctuations, optimal temperatures often shift below the mean and are accompanied by broader TPCs (Amarasekare and Johnson 2017; Amarasekare and Savage 2012; Gilchrist 1995; Deutsch et al. 2008; Martin and Huey 2008; Suleiman et al. 2025). Such adaptive responses resemble those observed in ectothermic species inhabiting temperate or polar regions (insects; Deutsch et al. 2008), where environmental temperatures regularly fluctuate within the lower bounds of their thermal tolerance range.

Under high-magnitude stochastic fluctuations, populations adapt to lower thermal extremes for peak competitive performance, giving rise to right-skewed J-shaped competition TPCs (red regions; Fig. 4). These asymmetric curves reflect a form of cold-specialist strategy in competition, where peak performance occurs near the lower thermal limits, indicating adaptation to colder environments. Such skewed performance curves are consistent with empirical observations (e.g., Toxopeus and Sinclair 2018) of freeze-tolerant insects inhabiting temperate, polar, and high-altitude regions, where cold specialisation allows survival under extreme and unpredictable thermal conditions. In these environments, insects rely on freeze tolerance or freeze avoidance, i.e., through physiological adaptations such as supercooling or behavioural mechanisms such as heightened motility and body vibrations (which can also enhance performance in competitive contests)**,** as key strategies enabling year-round persistence in harsh climates (Sinclair et al. 2003). In our model, this cold-specialist adaptation emerges as a right-skewed reaction norm specifically under high-magnitude stochastic fluctuations (${\sigma }_{\epsilon }>1$), highlighting behavioural adaptation to cold extremes under severe thermal unpredictability (Bale 1996; Sinclair 1999).

Our results underscore the significant role of environmental noise in shaping the evolutionary adaptation of thermal performance curves (TPCs), revealing that greater thermal variability promotes the selection of broader performance breadths characteristic of thermal generalists. This shift, marked by a leftward shift of the optimal temperature for peak competition and an expansion of the TPC breadth for active competition, emerges from the combined effects of selection on both the shape of competition TPCs. Empirical studies similarly report that temperate species often experience reduced physiological performance at average ambient temperatures due to environmental thermal variability (Vasseur et al. 2014). Our theoretical results suggest that such performance declines can be attributed to high-magnitude stochastic temperature fluctuations and the inherent trade-offs in competitive performance across thermal extremes (Angilletta 2009).

Temperature-dependent competitive interactions have been studied previously (e.g., Lax et al. 2020; Sunday et al. 2024). Empirical evidence indicates that such interactions can shift species’ optimal temperatures toward lower (cooler) values under interspecific competition (Sunday et al. 2024). In terms of evolutionary outcomes, their results are consistent with ours: their model focuses on interspecific competition, whereas ours considers intraspecific competition mediating density dependence. In Lax et al. (2020), the strength of competition between fast- and slow-growing species varies with temperature and is influenced by environmental factors such as mortality and resource supply. Their model predicts that slower-growing species are favoured at higher temperatures. In contrast, our results suggest that competition drives the TPC toward lower optimal temperatures for peak competitive performance, highlighting how distinct ecological contexts can yield divergent evolutionary responses to thermal variation.

### Model Assumptions and Limitations

It is well established that the Ricker model can exhibit a wide range of population dynamical behaviours (stable node, periodic oscillation and chaos) depending on the maximum population growth rate (May 1974). Similarly, the population dynamics in our model, though driven by temporal changes in temperature, displays comparable ecological patterns in the absence of environmental stochasticity (Figs. S1a–f), particularly when the intrinsic growth rate and carrying capacity are varied from their reference values (see Table 1). However, these different ecological behaviours observed in our model do not appear to affect the evolutionary dynamics of TPC shapes (Fig. S10).

To study the evolution of TPC shape in competition under stochastic environments, several simplifying assumptions were made. Specifically, we assumed that the beta-distributed TPC of competitive performance is independent of the intrinsic growth rate ($r$) and carrying capacity ($k$). In our model, all demographic rates—namely $r$, $k$, and competition coefficients—are temperature dependent. However, the focus of this study is the evolution of the TPC associated with competitive performance, where “performance” refers explicitly to the outcomes in competitive interactions. The temperature dependence of $r$ is modelled as a saturating exponential function, representing the TPC of $r$, while $k$ follows a bell-shaped temperature dependence, corresponding to the TPC of $k$. An optimum temperature can be defined for each TPC; for example, the optimum of the TPC of $k$ is given directly by ${\tau }_{m}$ in Eq. (4). Our analysis, therefore, focuses on the evolution of the TPC shape for competitive ability, while assuming that the TPCs of $r$ and $k$ remain fixed. Under this framework, traits such as speed, motility, aggression, and feeding rate are primarily interpreted as mediators of competitive interactions. We acknowledge that these traits may be multifunctional and can also influence $r$ and $k$, but including their joint evolution would substantially increase complexity and reduce interpretability. The evolution of the TPCs of $r$ and $k$ is an important topic within our broader research agenda but requires separate, dedicated analyses beyond the scope of the present study.

To remain consistent with the standard adaptive dynamics (AD), we assumed no intra-population variability in TPCs (i.e., the TPC represents either identical individual TPCs or the population average), and evolution of the shape of competition TPC follows the standard AD assumptions of rare, small-effect mutations acting independently on the TPC mean ($\mu$) and scaling parameter ($s$). Collectively, these assumptions produce adaptive patterns consistent with observations in temperate or polar ectotherms, and the results are robust to mutational covariance among TPC shape parameters (Figs. S11–12).

### Potential for Empirical Testing

The feasibility of experimental tests is inherently taxon dependent. In designing this study, we targeted the motility performance in ectotherms such as lizards and insects, because these traits have already been quantified in laboratory settings by exposing individuals to different thermal regimes and measuring multiple indices of locomotor performance (e.g., Angilletta 2009). Under the assumptions of our model, ratios of these performance indices among individuals can be interpreted as proxies for competitive interactions. In contrast, estimating demographic parameters such as the intrinsic population growth rate and carrying capacity for these taxa would require substantially longer and more involved experiments across multiple generations. This would entail either allowing individuals to reproduce and die to estimate intrinsic growth rates, or maintaining large groups under varying resource conditions to estimate carrying capacity.

Previous studies conducted under fluctuating environmental conditions (e.g., Jeremy and Peter 2001; Jiang and Morin 2004) have shown that the intrinsic growth rate and carrying capacity respond differently to temperature. In these studies, the intrinsic growth rate of some bacteria species peaked below 0.5, while mean population density in the absence of interspecific competition reached a maximum below 4 (unit scalable). Accordingly, the reference values adopted in this model are consistent with the reported ranges and yield sensible and testable outcomes. To enhance the real-world applicability of our theoretical model, we aim to adopt an empirical approach to test how fine-scale temporal thermal regimes (e.g., temperature time series obtained from thermal loggers along latitudinal gradients) influence win–loss ratios between individuals of different genotypes in controlled contest-arena experiments under diverse thermal conditions. We will also examine how competition TPC shapes of focal species vary along latitudinal thermal gradients.

Bacteria are widely recognised as alternative and biologically tractable model organisms. Competition experiments under fluctuating temperature regimes have been extensively conducted using microbial systems. For example, an experimental evolution study by Lambros et al. (2021) demonstrated that competition between *Escherichia coli* strains can lead to the evolution of generalist versus specialist strategies under periodically versus randomly fluctuating temperature regimes, respectively. In addition, several studies have examined adaptive evolution in single microbial species exposed to contrasting thermal or environmental selection regimes (e.g., Bennett et al. 1992; Bennett and Lenski 1993; Duncan et al. 2011; Laws and Belovsky 2010; Mallard et al. 2020). Further laboratory competition experiments to test this model across different thermal regimes could therefore be conducted using *E. coli*. This model organism offers substantial advantages for experimental evolution, including precise environmental control, the ability to define and test adaptation across a wide range of temperatures, replication of experimental lines, reciprocal assays at different selection temperatures, maintenance under well-defined culture conditions with large population sizes over thousands of generations, and propagation under distinct thermal regimes (Bennett et al. 1992; Jessup et al. 2004).

### Conclusion and Future Directions

In our previous study with periodic environments (Suleiman et al. 2025), evolution could lead to bimodal competition TPCs, which could be interpreted as coexistence of two phenotypes with high competitive performance at extreme temperatures. In the current study, such a scenario did not emerge, possibly because the added white noise might have increased the temperature variability in such a way that two distinct performance peaks do not emerge, but rather a wider single peak. In addition, although coexistence between two different competition TPCs is clearly possible, in our framework, different TPCs only arise through incremental evolution, so the TPCs of mutants and residents are close enough to not allow coexistence due to limiting similarity (this is the core of the framework of Adaptive Dynamics to track directional evolution through sequential mutant-resident substitutions), and evolutionary branching did not occur. Of course, gene flow from other areas could bring in phenotypes that are different but can still coexist with the residents, although we did not consider this scenario.

The model, grounded in density-dependent intraspecific competition under variable thermal environments, highlights how the periodicity and stochasticity of thermal fluctuations can influence the evolutionary outcome of TPC shapes in competition. Our findings demonstrate that while periodic environments with minimal stochasticity favour thermal specialists, stochastic environments consistently promote the evolution of generalist strategies. Furthermore, the results emphasise the importance of jointly considering both mean temperature and thermal variability when assessing the evolutionary consequences of climate change on population performance (Bozinovic et al. 2011; Estay et al. 2014; Vasseur et al. 2014). Full thermal specialisation and local adaptation, expressed through steep unimodal TPCs, evolve in stable thermal regimes. In contrast, broader unimodal and right-skewed J-shaped TPCs emerge as adaptive responses to more unpredictable and extreme thermal conditions. The evolution of the optimal temperature toward lower extremes for competitive performance, along with empirical evidence of freeze tolerance and avoidance as key strategies for survival in extreme and unpredictable thermal conditions, warrants further empirical testing. Interaction strength data reported by Sunday et al. (2024) and Lax et al. (2020) can be used to test the theoretical framework proposed here. Future studies could also expand the theoretical framework to explore more complex ecological scenarios involving multispecies interactions, multidimensional trait spaces, and more complex thermal regimes (e.g., coloured noise) to further elucidate the evolutionary dynamics of thermal adaptation in fluctuating environments.

## Supplementary Information

Below is the link to the electronic supplementary material.

Supplementary file1 (DOCX 4250 kb)

## Data Availability

MATLAB codes are available on https://github.com/abdulrahamanlawals-ship-it/AL_Suleiman.
