# Supplementary material for: Environmental Stochasticity Drives Adaptation to Cooler Thermal Optima in Competition
Source: Bull Math Biol. 2026 Mar 18;88(4):60. doi: 10.1007/s11538-026-01614-6 (PMC12999656; doi:10.1007/s11538-026-01614-6)
Supplement: Supplementary file 1 — Supplementary file1 (DOCX 4250 kb) [file 11538_2026_1614_MOESM1_ESM.docx]

# **SUPPLEMENTARY INFORMATION**

A. L. Suleiman, P. Landi & C. Hui. Environmental stochasticity drives adaptation to cooler thermal optima in competition.

## Appendix S1: The stochastic temperature dynamics

We model the stochastic temperature variations experienced by ectotherms in unpredictably fluctuating environments as a random environmental noise using the following mathematical function:

$\tau_{t}={(\tau}_{0}+m\sin\left( \omega t \right))S\left( \sigma_{\epsilon}X_{t} \right)$, $t\boldsymbol{\in}\mathbb{Z}^{+}$, (S1.1)

where $X_{t}$ is a standard normally distributed random variable with mean 0 and standard deviation of 1, written mathematically as $X_{t}\sim N(0,1)$, while $\sigma_{\epsilon}$ is the environmental noise magnitude. The sigmoid function $S\left( {\sigma_{\epsilon}X}_{t} \right)$ transforms the random variable ${\sigma_{\epsilon}X}_{t}$ from the unbounded range $(-\infty,\infty)$ to the bounded range $(0,1)$, as shown in Fig. S5a-d. It is defined as follows:

$S\left( \sigma_{\epsilon}X_{t} \right)=\frac{1}{1+\exp\left( -\sigma_{\epsilon}X_{t} \right)}$ ; (S1.2)

Substituting Eq. (S1.2) into Eq. (S1.1), we get:

$\tau_{t}=\frac{\left( \tau_{0}+m\sin\left( \omega t \right) \right)}{1+\exp\left( -\sigma_{\epsilon}X_{t} \right)}$ . (S1.3)

Here, $\tau_{t}$ represents the stochastic temperature dynamics obtained by combining the periodic temperature dynamics with the sigmoid function of the environmental noise $S\left( {\sigma_{\epsilon}X}_{t} \right)$. Fig. S5e-f displays such stochastic temperature dynamics for varying environmental noise magnitudes ($\sigma_{\epsilon}$).

## Appendix S2: Thermal performance curves (TPCs)

We model the thermal performance curves (TPCs) of ectotherms in stochastic environments using the beta probability density function. The TPC is expressed in reparametrized form as (Suleiman et al. 2025):

$\beta\left( \tau_{t};\mu,s \right)={\tau_{t}}^{\left( \frac{\mu}{s}-1 \right)}(1-\tau_{t})^{\left( \frac{1-\mu}{s}-1 \right)}\frac{\Gamma\left( \frac{1}{s} \right)}{\Gamma\left( \frac{\mu}{s} \right)\Gamma\left( \frac{1-\mu}{s} \right)}$; $\mu\in(0,1)$, $s\in(0,\infty)$. (S2)

Here, for a positive real number $z$, the gamma function is defined as $\Gamma\left( z \right)=\int_{0}^{\infty} x^{\left( z-1 \right)}e^{-x}dx$. In the above beta function, the ratio of gamma functions acts as a normalization factor to ensure the area under the performance curves is preserved ($=1$). The TPC shape is captured by the mean ($\mu$) and the scaling parameter ($s$). For biological reasons, the stochastic ambient temperature ($\tau_{t}$) is constrained to fluctuate between 0 and 1, which represent the critical thermal extremes (i.e., minimum and maximum) of the TPC. The scaling parameter controls the breadth of the TPC, influencing a generalist-specialist trade-off between itself and the peak performance of the curve (Angilletta 2009): a smaller scaling parameter produces narrower TPCs with higher peak performance, whereas a larger scaling parameter results in broader TPCs with lower peak performance (Fig. 1). The TPC determines individual performance for resource utilization/acquisition during contest competition ($\alpha$) between residents and mutants. The resources are captured by the carrying capacity *k*, which changes stochastically with temperature, but independently of the competition function ($\alpha$) and the beta distributed TPC.

## Appendix S3: Derivation of the invasion fitness and selection gradients in a stochastic environment.

From the stochastic Ricker competition model (Eq. 5), the per-capita growth rates of the residents $N_{t}$ and mutants $M_{t}$ in the stochastic thermal environment are computed as follows:

$\begin{matrix} f_{N}\left( N_{t},M_{t},\tau_{t} \right)=\exp\left( r\left( \tau_{t} \right)\left( 1-\frac{N_{t}+\alpha_{NM}\left( \tau_{t} \right)M_{t}}{k\left( \tau_{t} \right)} \right) \right) \\ f_{M}\left( N_{t},M_{t},\tau_{t} \right)=\exp\left( r\left( \tau_{t} \right)\left( 1-\frac{\alpha_{MN}\left( \tau_{t} \right)N_{t}+M_{t}}{k\left( \tau_{t} \right)} \right) \right) \end{matrix}$, (S3.1)

where $f_{N}\left( . \right)$ and $f_{M}\left( . \right)$ denote the per-capita growth rate of the residents and mutants respectively. Additionally, $N_{t}$ and $M_{t}$ represents the population size of the residents and mutants at time $t$, and $\tau_{t}$ represents the stochastic temperature dynamics (Eq. 1, S1.3). The competition coefficients, $\alpha_{MN}(\tau_{t})$ and $\alpha_{NM}\left( \tau_{t} \right)$, represent the competitive impact of residents on mutants and vice-versa at the prevailing temperature. In a fluctuating environment, the invasion fitness is defined as the long-term average per-capita growth rate of a mutant when rare ($M_{t}\to0$) within the resident population at its stationary distribution (Metz et al. 1992). The invasion fitness is computed using the expression for $f_{M}\left( . \right)$ in Eq. (S3.1) as:

$\bar{\lambda}(\mu_{N},s_{N}, \mu_{M},s_{M})= \lim_{L\to\infty}\frac{1}{L} \sum_{t=0}^{L} \left( r(\tau_{t})\left( 1-\frac{\alpha_{MN}(\tau_{t})N_{t}}{k(\tau_{t})} \right) \right)$ (S3.2)

In Eq. (S3.2), $L$ is the length of time range used to calculate the long-term average. From Eq. (S3.2), the selection gradients on the mean ($\bar{g}_{\mu}$) and scaling parameter ($\bar{g}_{s}$) of the TPC are derived as the partial derivatives of the long-term invasion fitness with respect to the mutant TPC shape $(\mu_{M}, s_{M})$, evaluated at the resident TPC shape $(\mu_{N}, s_{N})$. The bars over the invasion fitness $\lambda$ and selection gradients $g_{y}\left( y=\mu,s \right)$ indicate the long-term average. These quantities are expressed mathematically as:

$\begin{matrix} \bar{g}_{\mu}=\left. \frac{\partial\bar{\lambda}(\mu_{N},s_{N}, \mu_{M},s_{M})}{\partial\mu_{M}} \right|_{(\mu_{M}, s_{M})=(\mu_{N}, s_{N})} \\ \bar{g}_{s}=\left. \frac{\partial\bar{\lambda}(\mu_{N},s_{N}, \mu_{M},s_{M})}{\partial s_{M}} \right|_{(\mu_{M}, s_{M})=(\mu_{N}, s_{N})} \end{matrix}$.

The selection gradient of the TPC mean is:

$\bar{g}_{\mu}=\frac{\partial}{\partial\mu_{M}}\left. \left( \lim_{L\to\infty}\frac{1}{L} \sum_{t=0}^{L} \left( r(\tau_{t})\left( 1-\frac{\alpha_{MN}(\tau_{t})N_{t}}{k(\tau_{t})} \right) \right) \right) \right|_{(\mu_{M}, s_{M})=(\mu_{N}, s_{N})}$ (S3.3)

By differentiation of the series rule, the summation sign and the derivative operator can be interchanged. This simplifies Eq. (S3.3) to:

$\bar{g}_{\mu}=\left. \lim_{L\to\infty}\frac{1}{L} \sum_{t=0}^{L} \frac{\partial}{\partial\mu_{M}}\left( r(\tau_{t})-\frac{r(\tau_{t})\alpha_{MN}(\tau_{t})N_{t}}{k(\tau_{t})} \right) \right|_{(\mu_{M}, s_{M})=(\mu_{N}, s_{N})}$

$=\lim_{L\to\infty} \frac{1}{L}\sum_{t=0}^{L} \left( \frac{\partial}{\partial\mu_{M}}\left. \left( r\left( \tau_{t} \right) \right) \right|_{\left( \mu_{M}, s_{M} \right)=\left( \mu_{N}, s_{N} \right)} \right)-\lim_{L\to\infty}\frac{1}{L} \sum_{t=0}^{L} \left. \frac{r(\tau_{t})N(t)}{k(\tau_{t})}\left( \frac{\partial\alpha_{MN}(\tau_{t})}{\partial\mu_{M}} \right) \right|_{(\mu_{M}, s_{M})=(\mu_{N}, s_{N})}$ (S3.4)

To compute the selection gradient with respect to the mutant TPC mean $\mu_{M}$ as defined by Eq. (S3.4), we compute the derivative of the second term, since it is the term containing the performance indices. That is:

$\bar{g}_{\mu}=-\lim_{L\to\infty}\frac{1}{L} \sum_{t=0}^{L} \left. \frac{r(\tau_{t})N(t)}{k(\tau_{t})}\left( \frac{\partial\alpha_{MN}(\tau_{t})}{\partial\mu_{M}} \right) \right|_{(\mu_{M}, s_{M})=(\mu_{N}, s_{N})}$, (S3.5)

where $\alpha_{MN}\left( \tau_{t} \right)$ represents the competition coefficient of residents on mutants derived from Eq. (6) as:

$\alpha_{MN}\left( \tau_{t} \right)={\tau_{t}}^{\left( \frac{\mu_{N}}{s_{N}}-\frac{\mu_{M}}{s_{M}} \right)} \left( 1-\tau_{t} \right)^{\left( \frac{1-\mu_{N}}{s_{N}}-\frac{1-\mu_{M}}{s_{M}} \right)}\left( \frac{\Gamma\left( \frac{1}{s_{N}} \right)\Gamma\left( \frac{\mu_{M}}{s_{M}} \right)\Gamma\left( \frac{1-\mu_{M}}{s_{M}} \right)}{\Gamma\left( \frac{1}{s_{M}} \right)\Gamma\left( \frac{\mu_{N}}{s_{N}} \right)\Gamma\left( \frac{1-\mu_{N}}{s_{N}} \right)} \right)$.

The partial derivative of $\alpha_{MN}(\tau_{t})$ with respect to $\mu_{M}$ computed using logarithmic differentiation and evaluated at the resident performance indices is given as:

$\left. \frac{\partial\alpha_{MN}(\tau_{t})}{\partial\mu_{M}} \right|_{\begin{aligned} \mu_{M}=\mu_{N} \\ s_{M}=s_{N} \end{aligned}}=\ln\left[ \left( \frac{\tau_{t}}{1-\tau_{t}} \right)^{\frac{1}{s}} \right]+\frac{1}{s}\left( \psi\left( \frac{1-\mu}{s} \right)-\psi\left( \frac{\mu}{s} \right) \right)$ (S3.6)

Substituting the right-hand side of the first equation in Eq. (S3.6) into Eq. (S3.5) and simplifying, the selection gradient of the TPC mean $\bar{g}_{\mu}$ is:

$\bar{g}_{\mu}=\lim_{L\to\infty} \frac{1}{L}\sum_{t=0}^{L} \left( \ln\left[ \left( \frac{\tau_{t}}{1-\tau_{t}} \right)^{\frac{r\left( \tau_{t} \right)N_{t}}{k\left( \tau_{t} \right)s}} \right]+ \frac{r\left( \tau_{t} \right)N_{t}}{k\left( \tau_{t} \right)s}\left( \psi\left( \frac{1-\mu}{s} \right)-\psi\left( \frac{\mu}{s} \right) \right) \right)$ (S3.7)

The selection gradient of the TPC scaling parameter $\bar{g}_{s}$ is derived using the same approach as for the TPC mean, Eq. (S3.5):

$\bar{g}_{s}=\left. \lim_{L\to\infty}\frac{1}{L} \sum_{t=0}^{L} \frac{\partial}{\partial s_{M}}\left( r(\tau_{t})-\frac{r(\tau_{t})\alpha_{MN}(\tau_{t})N_{t}}{k(\tau_{t})} \right) \right|_{(\mu_{M}, s_{M})=(\mu_{N}, s_{N})}$

$=\lim_{L\to\infty} \frac{1}{L}\sum_{t=0}^{L} \left( \frac{\partial}{\partial s_{M}}\left. \left( r\left( \tau_{t} \right) \right) \right|_{\left( \mu_{M}, s_{M} \right)=\left( \mu_{N}, s_{N} \right)} \right)-\lim_{L\to\infty}\frac{1}{L} \sum_{t=0}^{L} \left. \frac{r(\tau_{t})N(t)}{k(\tau_{t})}\left( \frac{\partial\alpha_{MN}(\tau_{t})}{\partial s_{M}} \right) \right|_{(\mu_{M}, s_{M})=(\mu_{N}, s_{N})}$ (S3.8)

To compute the selection gradient with respect to mutants’ scaling parameter as defined by Eq. (S3.8), one computes the derivative of the second term which is a function of the performance indices. That is:

$\bar{g}_{s}=\lim_{L\to\infty}\frac{1}{L} \sum_{t=0}^{L} \left. \frac{r(\tau_{t})N(t)}{k(\tau_{t})}\left( \frac{\partial\alpha_{MN}(\tau_{t})}{\partial s_{M}} \right) \right|_{(\mu_{M}, s_{M})=(\mu_{N}, s_{N})}$ (S3.9)

The partial derivative of $\alpha_{MN}(\tau_{t})$ with respect to $s_{M}$ computed using logarithmic differentiation and evaluated at the resident performance indices is given as:

$\left. \frac{\partial\alpha_{MN}(\tau)}{\partial s_{M}} \right|_{\begin{aligned} \mu_{M}=\mu_{N} \\ s_{M}=s_{N} \end{aligned}}=\ln\left[ \left( \frac{\left( \tau\right)^{\mu}}{{(1-\tau)}^{(1-\mu)}} \right)^{\frac{1}{s^{2}}} \right]+\frac{1}{s^{2}}\left( \psi\left( \frac{1}{s} \right)-\mu\psi\left( \frac{\mu}{s} \right)-\left( 1-\mu\right)\psi\left( \frac{1-\mu}{s} \right) \right)$ (S3.10)

Substituting the right-hand side of the first equation in Eq. (S3.10) into Eq. (S3.9) and then simplifying, the selection gradient of the TPC scaling parameter $\bar{g}_{s}$ is:

$\bar{g}_{s}=\lim_{L\to\infty} \frac{1}{L}\sum_{t=0}^{L} \left( \ln\left[ \left( \frac{\left( \tau_{t} \right)^{\mu}}{\left( 1-\tau_{t} \right)^{\left( 1-\mu\right)}} \right)^{\frac{r\left( \tau_{t} \right)N\left( t \right)}{k\left( \tau_{t} \right)s^{2}}} \right]+\frac{r\left( \tau_{t} \right)N_{t}}{k\left( \tau_{t} \right)s^{2}}\left( \begin{aligned} \left( 1-\mu\right)\psi\left( \frac{1-\mu}{s} \right)+\mu\psi\left( \frac{\mu}{s} \right)- \\ \psi\left( \frac{1}{s} \right) \end{aligned} \right) \right)$ (S3.11)

From Eq. (S3.5) and Eq. (S3.11), the selection gradients with respect to the TPC mean and scaling parameter, which govern the evolution of the TPC shape are:

$\left( \begin{matrix} \bar{g}_{\mu} \\ \bar{g}_{s} \end{matrix} \right)=\left( \begin{matrix} \lim_{L\to\infty}\frac{1}{L} \sum_{t=0}^{L} \left( \ln\left[ \left( \frac{\tau_{t}}{1-\tau_{t}} \right)^{\frac{r\left( \tau_{t} \right)N_{t}}{k\left( \tau_{t} \right)s}} \right]+ \frac{r\left( \tau_{t} \right)N_{t}}{k\left( \tau_{t} \right)s}\left( \psi\left( \frac{1-\mu}{s} \right)-\psi\left( \frac{\mu}{s} \right) \right) \right) \\ \lim_{L\to\infty}\frac{1}{L} \sum_{t=0}^{L} \left( \ln\left[ \left( \frac{\left( \tau_{t} \right)^{\mu}}{\left( 1-\tau_{t} \right)^{\left( 1-\mu\right)}} \right)^{\frac{r\left( \tau_{t} \right)N_{t}}{k\left( \tau_{t} \right)s^{2}}} \right]+\frac{r\left( \tau_{t} \right)N_{t}}{k(\tau_{t})s^{2}}\left( \begin{aligned} \left( 1-\mu\right)\psi\left( \frac{1-\mu}{s} \right) \\ +\mu\psi\left( \frac{\mu}{s} \right)-\psi\left( \frac{1}{s} \right) \end{aligned} \right) \right) \end{matrix} \right)$ (S3.12)

## Appendix S4: Evolutionary stability analysis.

To assess the convergence stability of the evolutionary singularity $(\mu^{*},s^{*})$, we determine whether it is an attractor or a repeller of the evolutionary dynamics by calculating the sign of the eigenvalues of the Jacobian matrix ($J^{*}$) of the stochastic dynamical system (Eq. 9), evaluated at the singularity. This matrix is given by:

$J^{*}=\frac{1}{2}\hat{N}_{e}(\tau_{t})E\left( \begin{matrix} j_{\mu\mu} & j_{\mu s} \\ j_{s\mu} & j_{ss} \end{matrix} \right)$,

having elements

$j_{\mu\mu}=\left. \frac{\partial\bar{g}_{\mu}}{\partial\mu} \right|_{(\mu, s)=(\mu^{*}, s^{*})}$, $j_{\mu s}=\left. \frac{\partial\bar{g}_{\mu}}{\partial s} \right|_{(\mu, s)=(\mu^{*},s^{*})}$,

$j_{s\mu}=\left. \frac{\partial\bar{g}_{s}}{\partial\mu} \right|_{(\mu, s)=(\mu^{*}, s^{*})}$, $\left. j_{ss}=\frac{\partial\bar{g}_{s}}{\partial s} \right|_{(\mu, s)=(\mu^{*},s^{*})}$.

Since these expressions cannot be computed analytically, we used a numerical approach to investigate the convergence stability of the singularity when the mutational covariance matrix $E$ is computed as either the identity matrix or random matrix; note, $E$ is symmetric and positive definite. MATLAB software was used to calculate the eigenvalues of $J^{*}$ at the singular point $(\mu^{*}$, $s^{*})=(0.18,0.04)$, which is determined from the intersection of the nullclines of the dynamical system (Eq. 9). The eigenvalues of $J^{*}$are always real and negative. Both the singular point and its stability are independent of the mutational covariance matrix $E$ (Figs. S11-12). The singularity is therefore a convergence stable evolutionary attractor of the system (Eq. 9).

To assess the evolutionary stability of the singular point, we determine whether it is evolutionarily stable or unstable against the invasion of nearby mutant TPC shapes. This involves computing the eigenvalues of the Hessian matrix ($H^{*}$), which contains the second-order partial derivatives of the long-term invasion fitness for the mutant TPC shapes $(\mu_{M},s_{M})$, evaluated at the singularity. The Hessian matrix is given by:

$H^{*}=\left( \begin{matrix} h_{\mu\mu} & h_{\mu s} \\ h_{s\mu} & h_{ss} \end{matrix} \right)$,

with elements

$$\begin{matrix} h_{\mu\mu}=\left. \frac{\partial^{2}\bar{\lambda}}{\partial\mu_{M}^{2}} \right|_{(\mu_{M}, s_{M})=(\mu^{*}, s^{*})}, & {h_{\mu s}=\left. \frac{\partial^{2}\bar{\lambda}}{\partial\mu_{M}s_{M}} \right|}_{(\mu_{M}, s_{M})=(\mu^{*}, s^{*})}, \\ h_{s\mu}=\left. \frac{\partial^{2}\bar{\lambda}}{\partial s_{M}\partial\mu_{M}} \right|_{(\mu_{M}, s_{M})=(\mu^{*}, s^{*})}, & h_{ss}=\left. \frac{\partial^{2}\bar{\lambda}}{\partial s_{M}^{2}} \right|_{(\mu_{M}, s_{M})=(\mu^{*},s^{*})}. \end{matrix}$$

The singular point is evolutionarily stable if $H^{*}$ is negative definite, meaning all eigenvalues of $H^{*}$ evaluated at that point have negative real parts. We numerically tested the sign of the eigenvalues of $H^{*}$ with respect to the singular point $(\mu^{*}$, $s^{*})=(0.18,0.04)$ computed from the intersection of the nullclines of the dynamical system (Eq. 9). The results confirm that all the eigenvalues of $H^{*}$ have negative real parts, indicating that the singularity represents a fitness maximum.

From the analysis above, the singularity of the system (Eq. 9) is convergence stable and an evolutionarily stable strategy (ESS), thus a continuously stable strategy (CSS; Eshel 1983). A singularity that is both ESS-stable and convergence stable is termed a continuously stable strategy (CSS; Eshel 1983), indicating the endpoint of the evolutionary process.

## Supplementary figures


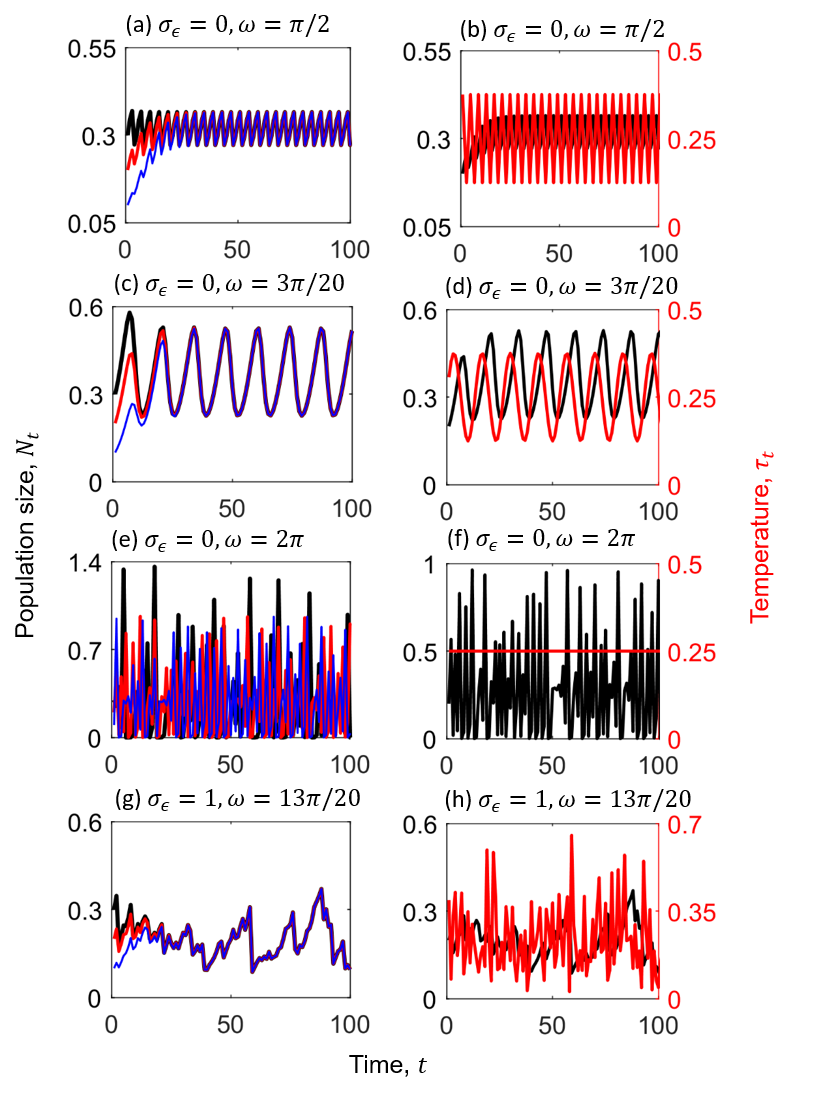


**Fig. S1** Effects of temperature fluctuations (Eq. 1) on the resident population dynamics (Eq. 2). In the absence of environmental noise, the population dynamics from three different initial conditions ($N_{0}=$ 0.3, 0.2, 0.1; shown in black, red and blue, respectively) exhibit different behaviours (regular steady-state, **a-b**; periodic, **c-d**; and chaotic, **e-f**) under periodic (red lines in panels **b** and **d**) and constant temperature regimes (red line, panel **f**). Temperature dynamics (red line; right y-axis) are shown in the absence of environmental noise (panels **b, d**, and **f**). (**g-h**) Under the influence of environmental noise, the population dynamics (black line, panel **g**) exhibit a long-term oscillating behaviour, driven by stochastic temperature dynamics (red line, panel **h**) with intermediate environmental noise ($\sigma_{\epsilon}=1$). In panels **g-h**, the initial conditions lose influence within the first 100 time steps. The corresponding population dynamics (black lines, left y-axis) in the absence of environmental noise (panels **b, d**, and **f**) differ in maximal values compared to the dynamics under the influence of environmental noise (panel **h**). Parameters: (**a-d, g-h**) $r_{0}=c=r_{1}=0.1$, $k_{0}=2$ (**e-f**) $r_{0}=5, c=r_{1}=0.1, k_{0}=1$. Other parameters: $\tau_{0}=0.5$, $m=0.25$, and $\sigma_{k}=0.05$. The list of model parameters is provided in Table 1


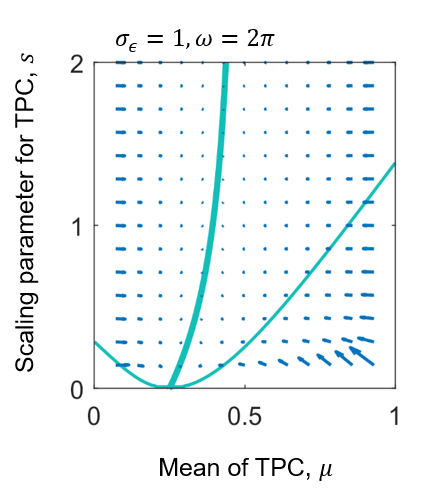


**Fig. S2** Evolutionary vector fields of Eq. (9) in the absence of environmental noise. Arrows show the magnitude and direction of evolutionary change. The bold curve represents the nullcline for the selection gradient of the TPC mean ($\bar{g}_{\mu}=0$), and the thin curve represents the nullcline for the TPC scaling parameter ($\bar{g}_{s}=0$). The singular point, computed numerically from the intersection of the nullclines of the dynamical system under the influence of chaotic ecological dynamics, is $(\mu^{*},s^{*})=(0.25,0)$. The singularity is continuously stable (i.e., convergence stable, meaning the evolutionary dynamics converge towards it, and evolutionarily stable, indicating the final endpoint of the evolutionary process). In this figure, the mutational covariance matrix $E$ is taken to be the identity matrix. Parameters: $r_{0}=5,$ $c=r_{1}=0.1,$ $k_{0}=1$, $\tau_{0}=0.5$, $m=0.25$, and $\sigma_{k}=0.05$. The list of model parameters is provided in Table 1


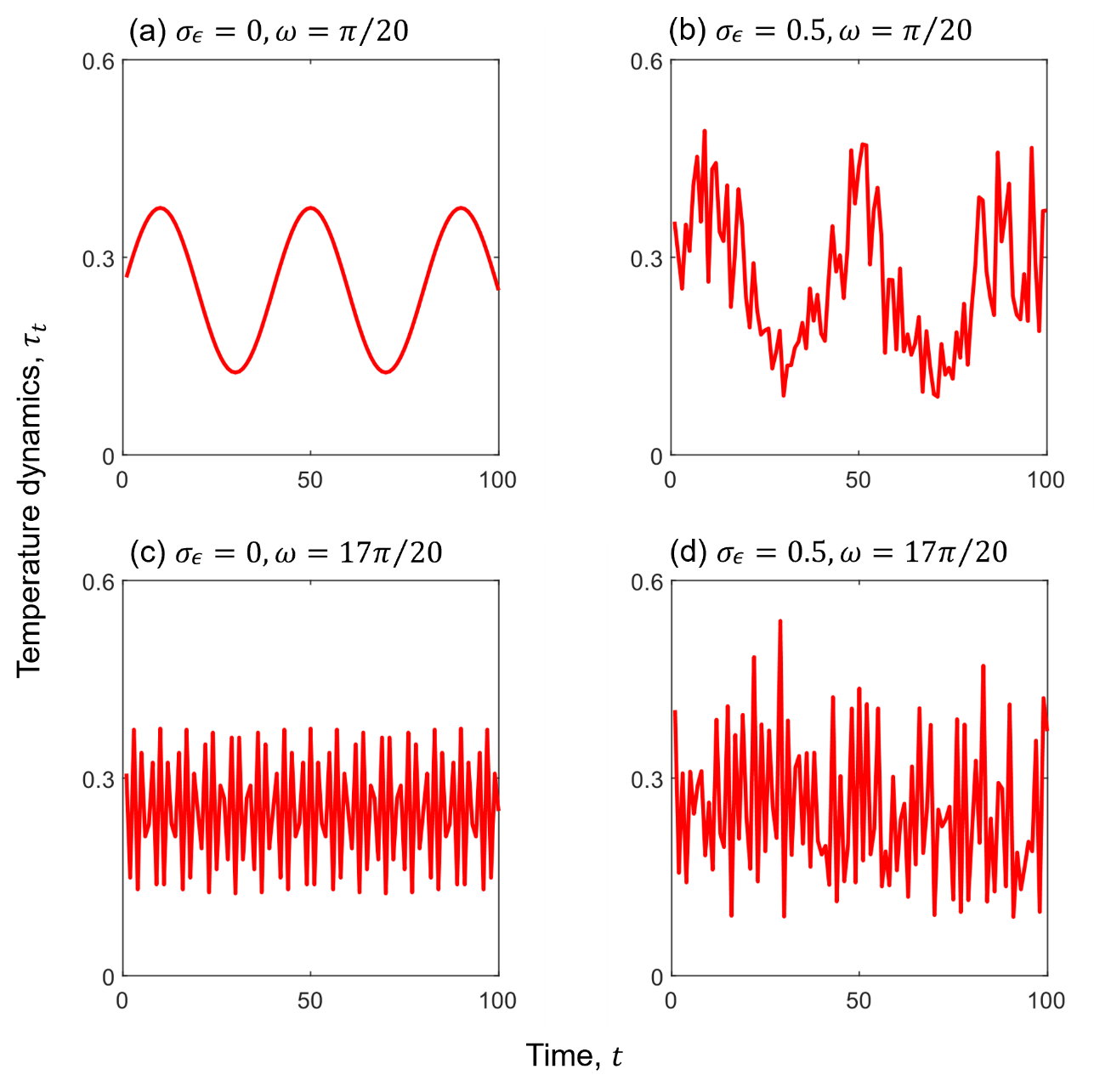


**Fig. S3** Temperature dynamics for varying noise magnitudes and fluctuation frequencies. The temperature dynamics exhibit periodic fluctuations when $\omega$ is a rational multiple of $\pi$ without stochasticity ($\sigma_{\epsilon}=0$), for instance, under slow fluctuations ($\omega=\pi/{20}$; panel **a**) and fast fluctuations ($\omega={17\pi}/{20}$; panel **c**). With low-magnitude noise ($\sigma_{\epsilon}=0.5$), the temperature dynamics become random under both slow ($\omega=\pi/{20}$; panel **b**) and fast fluctuations ($\omega={17\pi}/{20}$; panel **d**). Parameters: $\tau_{0}=0.5$ and $m=0.25$. The list of all model parameters is provided in Table 1


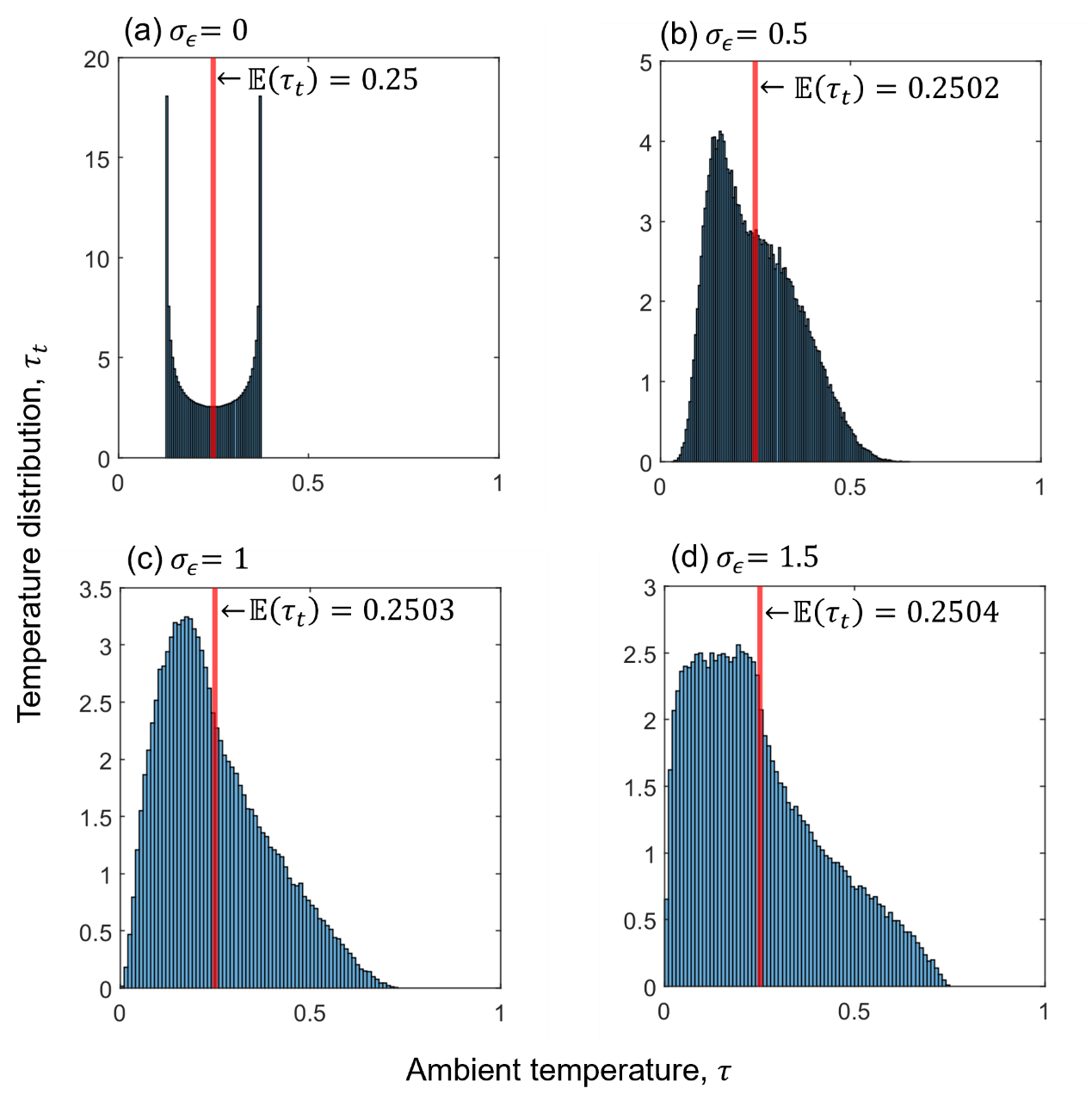


**Fig. S4** Histogram of the stochastic temperature dynamics and its mean. The figure shows the temperature distribution displayed as a normalised probability distribution under different environmental noise magnitudes ($\sigma_{\epsilon}$). The temperature distribution is sampled over 20,000 time steps. The red vertical line indicates the estimated sample mean for each distribution, which remains close to ${\tau_{0}}/2=0.25$. Parameters: $\tau_{0}=0.5$, $m=0.25$, and $\omega=1$. The list of all model parameters is provided in Table 1


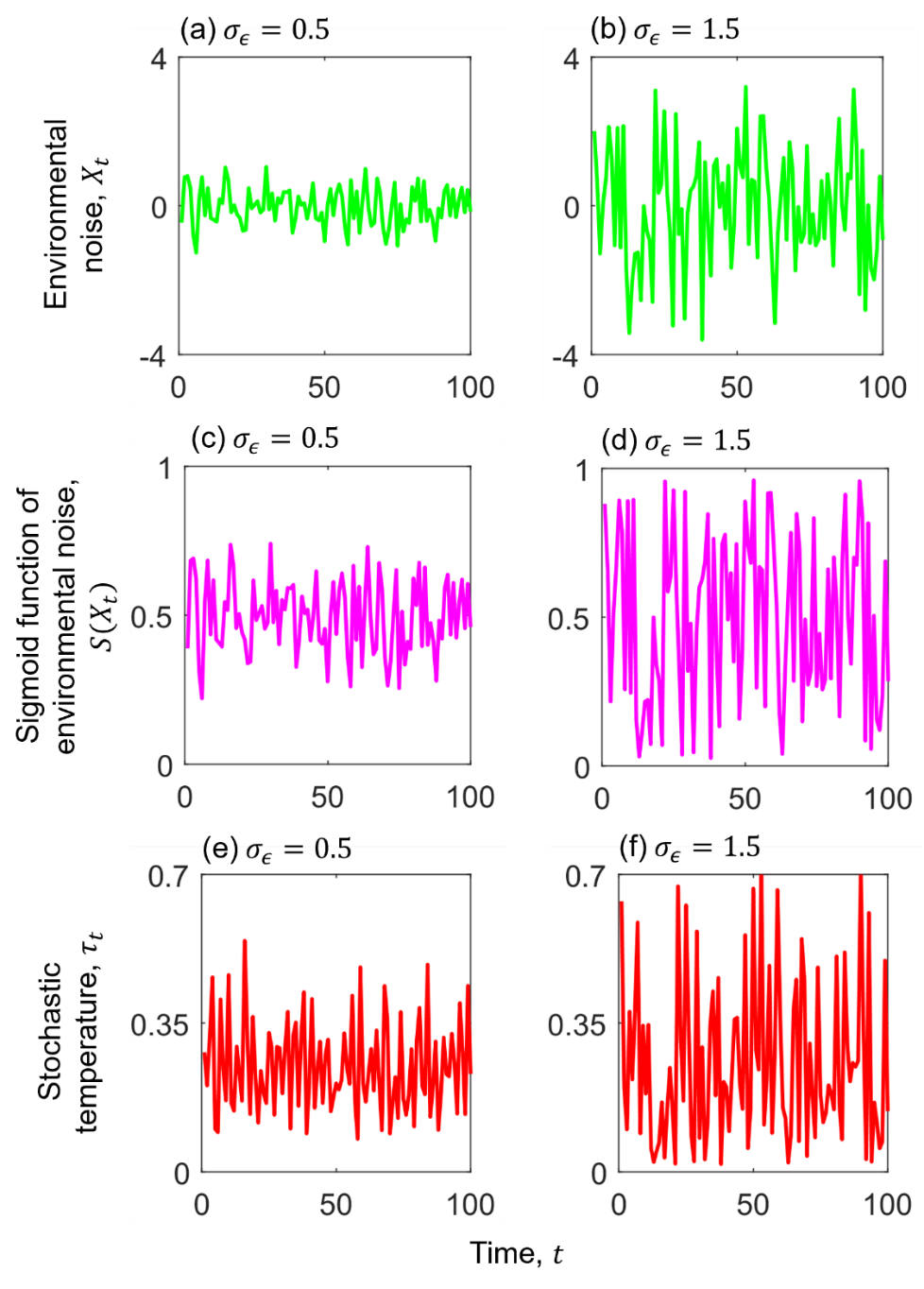


**Fig. S5** Environmental noise, its sigmoid function and stochastic temperature dynamics. (**a-b**) Environmental noise ${\sigma_{\epsilon}X}_{t}$ with mean 0 for different magnitudes of the noise $\sigma_{\epsilon}$. (**c-d**) The sigmoid function $S\left( {\sigma_{\epsilon}X}_{t} \right)$ transforms the environmental noise ${\sigma_{\epsilon}X}_{t}$ from the unbounded range $(-\infty,\infty)$ to the bounded range $(0,1)$. (**e-f**) Stochastic temperature dynamics (Eq. 1, S1.3), generated by multiplying the discrete sinusoidal temperature fluctuations with the sigmoid function of the environmental noise. The left panel shows the dynamics under low-magnitude noise ($\sigma_{\epsilon}=0.5$), while the right panel shows the dynamics under high-magnitude noise ($\sigma_{\epsilon}=1.5$). Parameters: $\tau_{0}=0.5$, $m=0.25$, and $\omega={13\pi}/{20}$. The list of all model parameters is provided in Table 1


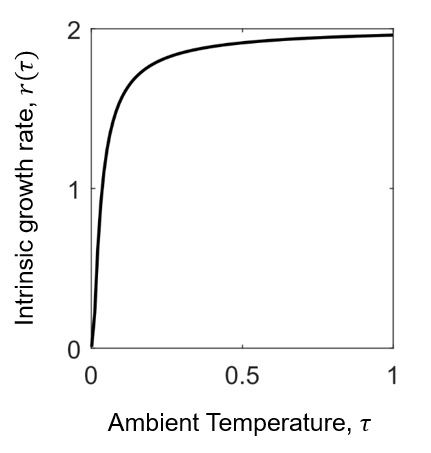


**Fig. S6** Intrinsic growth rate as a function of ambient temperature, $\tau$. The growth rate is low at near zero (critical minimum temperature), increases exponentially, and saturates as the temperature increases to one (critical maximum temperature). Parameters: $r_{0}=2$, $c=0.025$, and $r_{1}=0.01$.The list of all model parameters is provided in Table 1


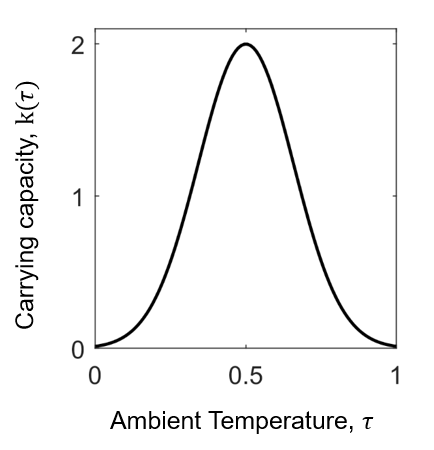


**Fig. S7** Carrying capacity as a function of the ambient temperature, $\tau$. The carrying capacity is modelled as a Gaussian function with low resource abundance at the critical thermal limits (0 and 1) and a peak at the optimal temperature for the carrying capacity, $\tau_{m}=0.5$. Parameters: $k_{0}=2$ and $\sigma_{k}=0.05$. The list of all model parameters is provided in Table 1


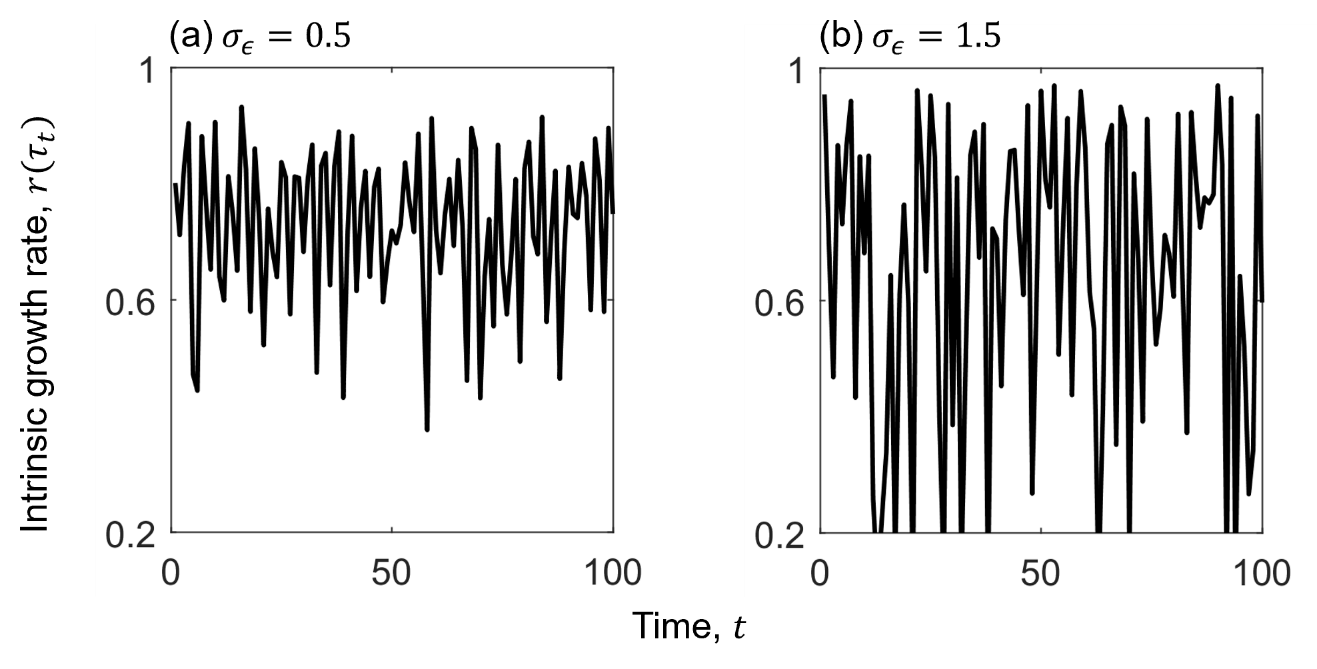


**Fig. S8** Influence of stochastic temperature dynamics (Eq. 1) on the intrinsic growth rate (Eq. 3). The intrinsic growth rate fluctuates stochastically under both low-magnitude ($\sigma_{\epsilon}=0.5$; panel **a**) and high-magnitude noise ($\sigma_{\epsilon}=1.5$; panel **b**). The growth rate’s variability becomes larger under high-magnitude noise compared to low-magnitude noise. Parameters: $r_{0}=c=r_{1}=0.1$, $\tau_{0}=0.5$, $m=0.25$, and $\omega={13\pi}/{20}$. The list of all model parameters is provided in Table 1


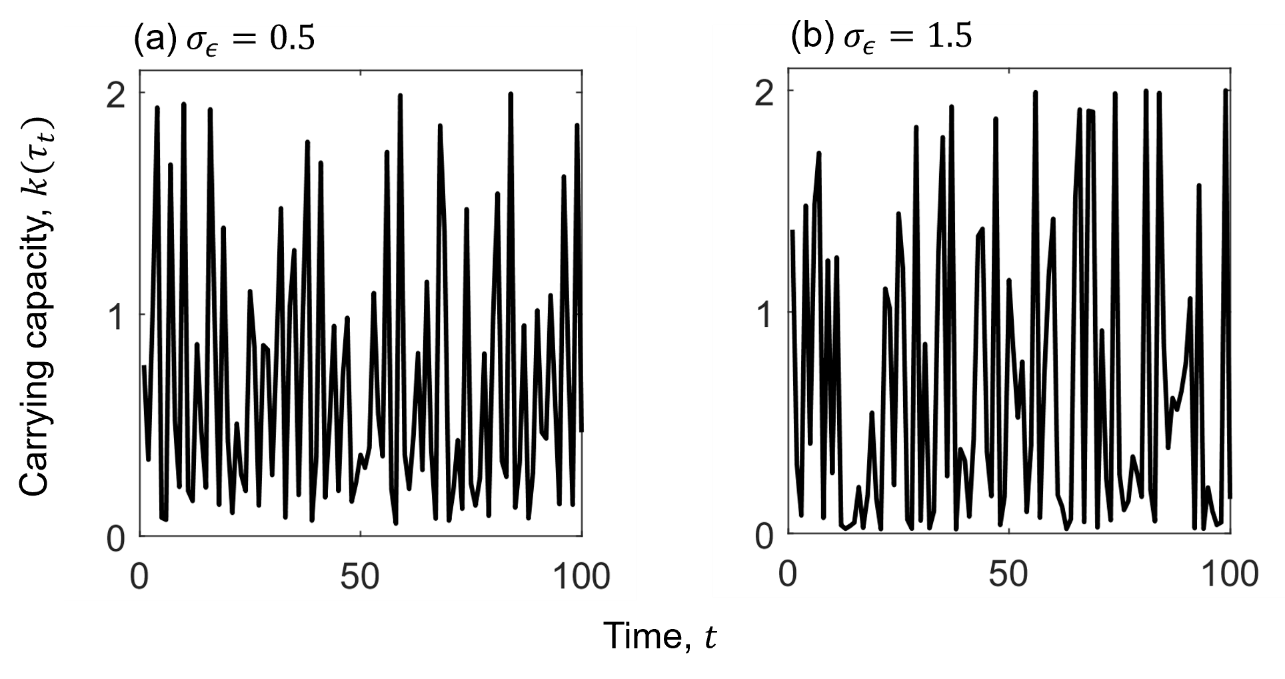


**Fig. S9** Influence of stochastic temperature dynamics (Eq. 1) on the carrying capacity (Eq. 4). The carrying capacity fluctuates stochastically when subjected to both low-magnitude ($\sigma_{\epsilon}=0.5$; panel **a**) and high-magnitude noise ($\sigma_{\epsilon}=1.5$; panel **b**), reaching its peak ($k_{0}=2$) in both cases. Parameters: $\tau_{0}=0.5$, $m=0.25$, $\omega={13\pi}/{20}$ and $\sigma_{k}=0.05$. The list of all model parameters is provided in Table 1


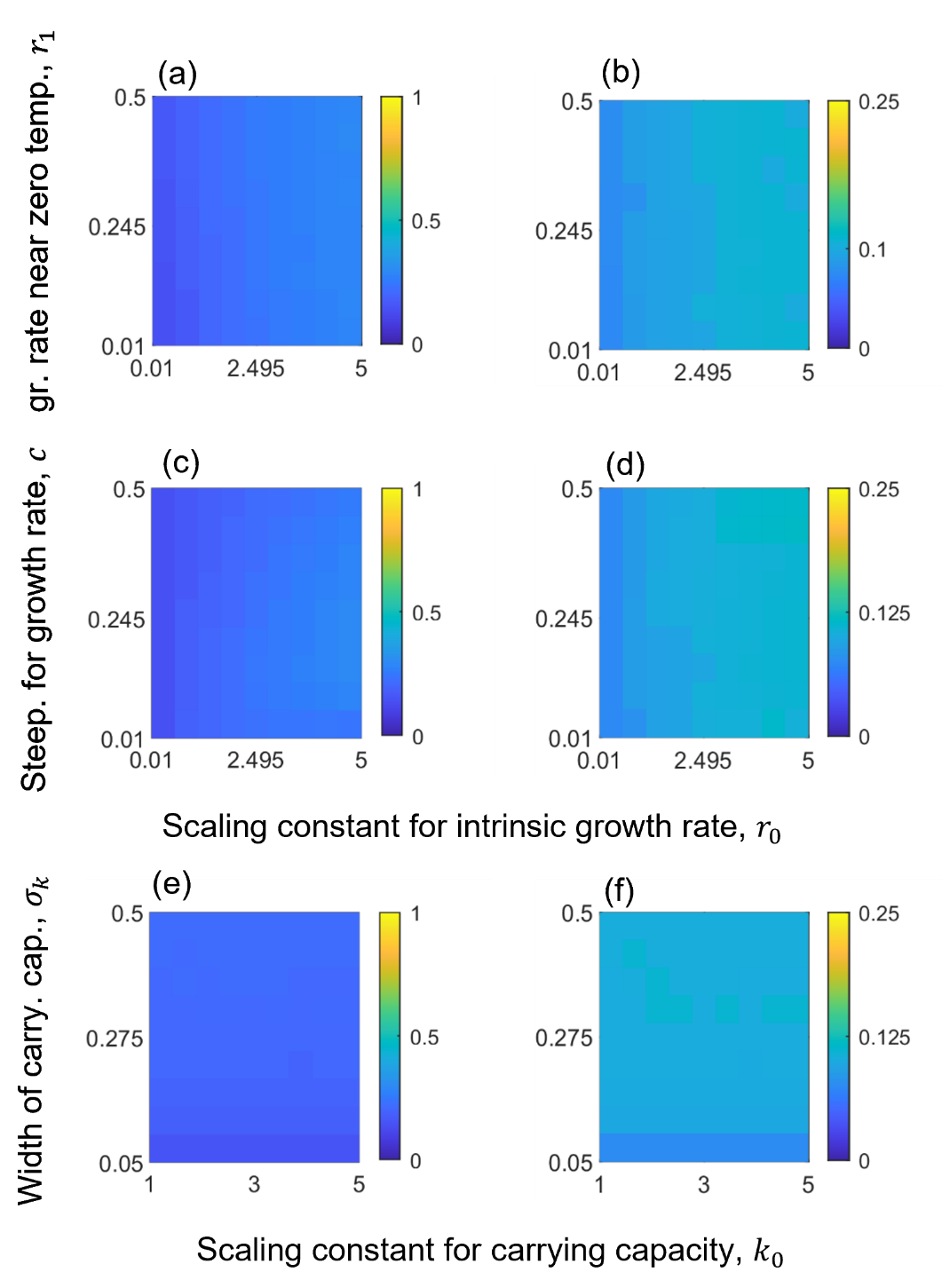


**Fig. S10** Impact of the scaling constant for the intrinsic growth rate $r_{0}$, the steepness of the growth rate $c$, the baseline growth rate near zero temperature $r_{1}$, the scaling constant for the carrying capacity $k_{0}$, and the width of the carrying capacity $\sigma_{k}$ on the evolutionarily stable strategy (ESS) of the TPC. Each pair of plots (panels **a-b**, **c-d**, and **e-f**) shows the evolutionary changes in the unimodal TPC through changes in the optimal TPC temperature ($\tau_{\mathrm{opt}}$) and the standard deviation (𝜎) regulating the TPC breadth. The ESS remains robust to changes in these parameters. Parameters: (**a-b**) $c=0.1$ and (**c-d**) $r_{1}=0.1$ with fixed $k_{0}=1$ and $\sigma_{k}=0.05$. Other parameters: $\tau_{0}=0.5$, $m=0.25$, $\sigma_{\epsilon}=0.5$, and$\omega={13\pi}/{20}$


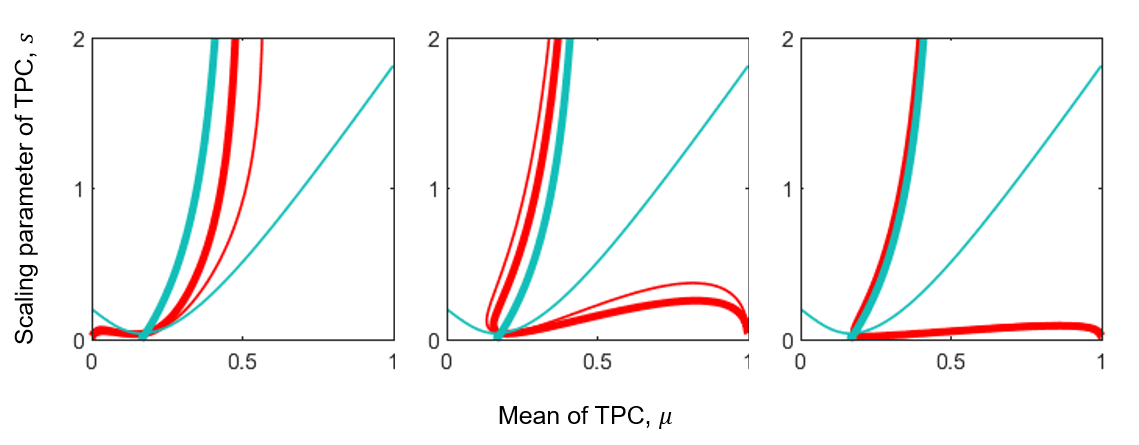


**Fig. S11** Effects of the identity and non-identity random mutational covariance matrices $E$ on the nullclines of the dynamical system (Eq. 9). Cyan curves represent the nullclines computed with the identity matrix, while the red curves indicate the nullclines computed with the non-identity random matrices. The bold curves represent the nullclines for the TPC mean (i.e., $\dot{\mu}=0$) and the thin curves represent the nullcline for the TPC scaling parameter (i.e., $\dot{s}=0$). With the identity and random matrices, the nullclines differ in shape, but intersect at the same singular point $(\mu^{*}$, $s^{*})=(0.18,0.04)$. Parameters: $r_{0}=c=r_{1}=0.1$, $k_{0}=2$, $\tau_{0}=0.5$, $m=0.25$, $\sigma_{\epsilon}=0.5$,$\omega={13\pi}/{20}$ and $\sigma_{k}=0.05$. The list of all model parameters is provided in Table 1


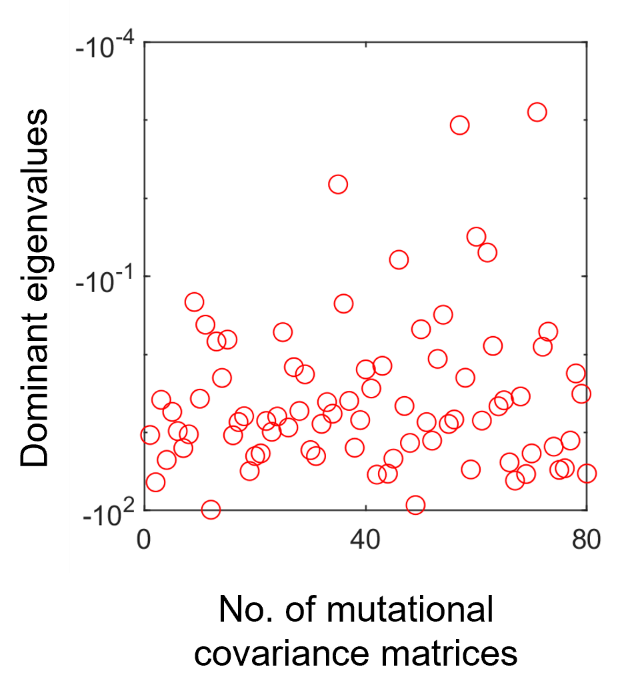


**Fig. S12** Dominant eigenvalues of the Jacobian matrix of the dynamical system. The eigenvalues are computed using 80 random mutational covariance matrices. Results show that the eigenvalues remain real and negative, indicating local stability. Parameters: $r_{0}=c=r_{1}=0.1$, $k_{0}=2$, $\tau_{0}=0.5$, $m=0.25$, $\sigma_{\epsilon}=0.5$,$\omega={13\pi}/{20}$ and $\sigma_{k}=0.05$. The list of all model parameters is provided in Table 1
